# Improving the performance and interpretability on medical datasets using graphical ensemble feature selection

**DOI:** 10.1093/bioinformatics/btae341

**Published:** 2024-06-05

**Authors:** Enzo Battistella, Dina Ghiassian, Albert-László Barabási

**Affiliations:** Network Science Institute, Northeastern University, Boston, MA 02115, United States; Scipher Medicine, Waltham, MA 02453, United States; Network Science Institute, Northeastern University, Boston, MA 02115, United States; Department of Data and Network Science, Central Eastern University, Budapest 1051, Hungary; Department of Medicine, Brigham and Women’s Hospital, Harvard Medical School, Boston, MA 02115, United States

## Abstract

**Motivation:**

A major hindrance towards using Machine Learning (ML) on medical datasets is the discrepancy between a large number of variables and small sample sizes. While multiple feature selection techniques have been proposed to avoid the resulting overfitting, overall ensemble techniques offer the best selection robustness. Yet, current methods designed to combine different algorithms generally fail to leverage the dependencies identified by their components. Here, we propose Graphical Ensembling (GE), a graph-theory-based ensemble feature selection technique designed to improve the stability and relevance of the selected features.

**Results:**

Relying on four datasets, we show that GE increases classification performance with fewer selected features. For example, on rheumatoid arthritis patient stratification, GE outperforms the baseline methods by 9% Balanced Accuracy while relying on fewer features. We use data on sub-cellular networks to show that the selected features (proteins) are closer to the known disease genes, and the uncovered biological mechanisms are more diversified. By successfully tackling the complex correlations between biological variables, we anticipate that GE will improve the medical applications of ML.

**Availability and implementation:**

https://github.com/ebattistella/auto_machine_learning.

## 1 Introduction

The ability of machine learning (ML) to detect novel disease-specific biomarkers from patterns in the data has the potential to revolutionize medicine. Yet, its predictive power is often limited by the high number of variables, also called features, compared to the small number of available training samples (Drucker and Krapfenbauer 2013). This can lead to overfitting, representing an outcome too specific to a dataset, offering biased and unreliable conclusions (Hawkins 2004). Acceptance by physicians also requires the interpretability of the predictions. Hence, dimensionality reduction methods (Guyon and Elisseeff 2003, Chandrashekar and Sahin 2014, Pepke and Ver Steeg 2017) have been proposed to identify a small number of biomarkers to use for classification. These techniques rely on statistical analysis (Bailey *et al.* 2018), classifiers (Li *et al.* 2017), and expert knowledge (Liu *et al.* 2020) to identify the most informative variables.

The low sample size can also lead to an unstable feature selection defined by an outcome specific to the experimental settings (He and Yu 2010). To increase robustness (Qayyum *et al.* 2021), ensemble approaches combine the strengths of several feature selection components, improving the results’ stability and accuracy (Saeys et al. 2008, Parvandeh *et al.* 2020) through a more thorough exploration of the space of possible selections (Bolón-Canedo and Alonso-Betanzos 2019). While ensemble approaches have been used for feature selection in the past, they have often been limited to simple techniques such as majority or weighted voting (Caba *et al.* 2021), hill climbing (Torgo and Gama 1996), ablation (Battistella 2021), or genetic algorithms (Tsymbal *et al.* 2005). By overlooking the synergistic effect of the features in each feature selection component, these approaches might break the complementarity of features selected by different components and introduce redundant information.

Graphs are a powerful tool to capture intricate relations within high-dimensional data. They have been used in various applications, including medical network analysis (Barabási *et al.* 2011) and ML approaches such as Graph Neural Networks (Scarselli *et al.* 2008). Despite their potential, the use of graph theory as an ensemble method has been limited. Most of the methods focus on feature selection on natural graph structures in the data (Rakhimberdina *et al.* 2020) or similarity-based graphs (Hashemi *et al.* 2020, Joodaki *et al.* 2021, Chamlal *et al.* 2022).

In this work, we introduce Graphical Ensembling (GE), a novel application of graph theory to ensemble feature selection approaches exploiting the relations between features highlighted by different learners, enabling us to tackle both overfitting and performance robustness. As we show, the method enhances relevance and eliminates redundancy within the selection while being highly adaptable and modular. To evaluate the proposed approach, we conducted extensive experiments on four medical datasets covering rheumatoid arthritis (RA), cancer, Covid-19, and myocardial infarction (MI), characterized by a wide range of data types, number of classes, balance between the classes, and variable-to-sample ratios. We compare the performance of our approach to baseline and task-specific feature selection approaches using a systematic classification framework to ensure a fair comparison. The relevance of our automatic pipeline is tested against a referential autoML method (Le *et al.* 2020). Finally, we show that GE represents a unique method relying on graph theory for assembling a minimal set of complementary features from diverse selections of features.

## 2 Methods

### 2.1 Overview of graphical ensembling

GE methods for feature selection leverage multiple feature selections on multiple folds of cross-validation to characterize the complementarity of features for a given task. The information on correlations of groups of features gathered by graphical ensemble methods corresponds to multi-collinearity information. To exploit this complex information, we introduce the concept of *co-selection graph* and its generalization, the *co-importance graph*. In these graphs, nodes represent features, and links are weighted by the number of times the features have been selected together in the case of the co-selection graph, or by the co-importance weights in the case of the co-importance graph. The co-importance weight is defined as the aggregation of the importance weights the various feature selection techniques attribute to the features. The higher the weight, the more relevant to the task the pair of features is.

#### 2.1.1 k-Heavy and k-W Heavy consensus feature selection

We propose the concept of *k-Heavy Consensus Feature Selection* (k-Heavy) and its extension on the co-importance graph, the *k-Weighted Heavy Consensus Feature Selection* (k-W Heavy) for ensemble feature selection. These methods use the notion of the k-heaviest subgraph (Letsios *et al.* 2016), which corresponds to the subgraph of *k* nodes presenting the highest sum of edge weights. We consider several feature selection techniques applied over multiple cross-validation splits. For a pair of features (*i*, *j*) and a feature selection technique *f*, let $M_{i,j}^{f}$ be the number of splits over which *f* selected both *i* and *j* simultaneously. We establish a co-selection matrix *M* where $M_{i,j}$ is the sum over all the feature selection methods *f* of $M_{i,j}^{f}$. In most ensemble feature selection methods, only the total number of selections of a given variable is used, i.e. $M_{i,i}$. We assume and prove in this paper that the other components of the co-selection matrix are valuable, indicating that each feature selection method proposes a selection of complementary variables with a low redundancy on every split. Then, the proposed approach aims to aggregate this knowledge over all splits and selection techniques to extract a set of robust features maximizing the co-selection and still verifying the complementarity and redundancy-free properties.

To leverage this co-selection information, we rely on an algorithm from graph theory and represent the matrix by a graph G=(V,E,w) where the nodes *V* are the features and the edges *E* are weighted by the function *w* such as for $e=(i,j)\in E,\;w(e)=M_{i,j}$. Our co-selection maximization objective will be translated as selecting the subset of *k* nodes *N* maximizing the sum of the edges’ weights in the graph induced by *N*, which corresponds to the well-studied Heaviest k-Subgraph problem. This NP-hard problem can be solved exactly or approximately through a branch and bound approach as proposed in Letsios *et al.* (2016). We applied their exact resolution method. However, the proposed pipeline could be sped up using the approximate one in case of a sizable co-selection graph due to the high dimensionality of the data and lowly constraining feature selection techniques.

As an extension of this approach, we rely on the importance weights of the feature selection techniques to build a co-importance graph. We denote I(f,s,i) the importance weight of feature *i* on split *s* according to the feature selection method *f*. The weight of edge *e* between feature *i* and feature *j* is defined as:

$$w(e)=\sum_{s,f}m\text{in}(I(f,s,i),I(f,s,j)).$$

The co-importance of (*i*, *j*) according to the feature selection technique *f* is defined as the minimum importance *f* grants to *i* and the one it grants to *j*. Intuitively, the weight of a feature on fold *i* with feature selection technique *f* is seen as a capacity. Thus, co-importance corresponds to the most limiting capacity between two features.

### 2.2 Systematic comparison pipeline

**Figure 1.**
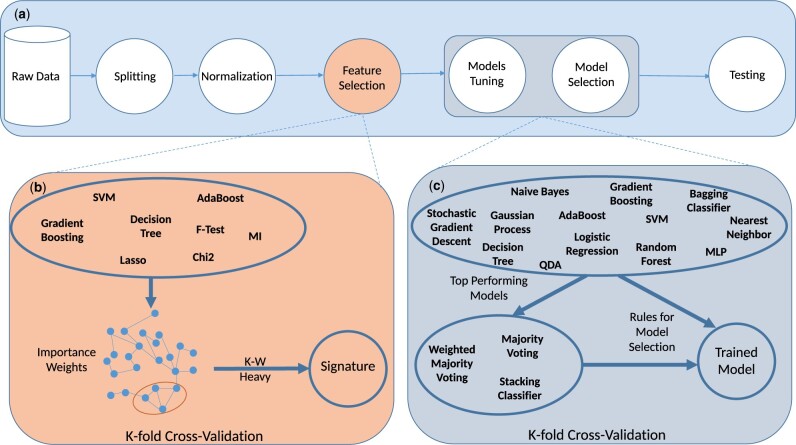
Proposed k-W Heavy pipeline. An overview of the different steps of the proposed feature selection and classification approach. (a) After data splitting and normalization, we perform feature selection. (b) We use the eight selection methods listed in the figure to define a matrix of co-importance weights. We define the co-importance graph whose nodes are the features and edges are weighted by the co-importance weights. Then, the proposed Graphical Ensembling feature selection technique enables us to identify the signature of variables. (c) In this signature’s space, we train the 13 considered classification models, and select the best-performing ones to build 3 ensemble models. Finally, we select the model reporting the best overall performance for testing using the proposed model selection rules.

We propose an automatic classification pipeline adapted from Chassagnon *et al.* (2021) to perform feature selection, classification model tuning, and selection (see Fig. 1). This framework enables a fair comparison of the different ensemble feature selection methods through standardization of the feature selection and the classification steps. We provide new rules for selecting the best classification model, formalized in Supplementary Material S0.3, to improve the fairness of comparison through better automatization. These rules rely on combinations of the different classification metrics and enforce the average training and validation performance over the different cross-validation folds to be within a chosen threshold (sanity constraint) while selecting the model with the best-averaged validation performance (efficiency constraint). The sanity constraint is used to prevent overfitting, which is often characterized by too high training performance compared to the validation. The efficiency constraint aims at maximizing the overall performance. Performances are then evaluated on an unseen test set.

#### 2.2.1 Classification framework

The main steps of the pipeline are:

1. Preprocessing: The data is normalized after the splitting using only the training set to avoid leaking information from the test set on the training set.
2. Feature Selection: We apply several feature selection methods on cross-validation splits. This approach is flexible and can leverage any selection technique, whether statistical or ML-based. We employed seven techniques focusing on varying data properties (details are provided in Supplementary Material S0.13).
3. Graphical Ensemble Feature Selection.
4. Classifier Training and Prediction: The resulting signature from the ensemble feature selection is used to train classifiers and perform prediction. We used 15 classifiers along with 3 ensemble methods (details are provided in Supplementary Material S0.13).
5. Model Selection: The final model is selected using rules as defined in Supplementary Material S0.3. These rules take into account multiple classification metrics and can favor different properties in the predictions.
6. Evaluation: Finally, the best model is assessed on the unseen test set.

### 2.3 Tasks and baseline methods

We conducted experiments on four datasets with varying numbers of samples, dimensionalities, data types, and tasks (see Table 1):

**Table 1.** Description of the data and tasks considered.

Dataset	Prediction	Data type	#Variables	#Patients	#Mild cases	#Severe cases
RA-MAP	RA severity	PBMCs + Clinical	17 817	227	126	101
Covid-19	Covid severity	Imaging + Clinical	543	693	554	139
MI	Complications	Metabolite + Clinical	111	1700	663	1037
TCGA	Cancer type (24 classes)	RNAseq	20 531	7045	NA	NA

We consider four medical datasets offering different challenges. RA-MAP has few samples relative to the high number of features. Covid-19 presents an important unbalance between the two classification classes. MI has many samples and few noisy variables. TCGA includes 24 classes and an important number of samples and variables.

- RA-MAP (Cope *et al.* 2017): The dataset includes 227 samples of patients suffering from Rheumatoid Arthritis (RA) for which gene expression in peripheral blood mononuclear cells covers 17 817 genes, and 30 clinical variables (Supplementary Material S0.13). The task considered on this dataset is patient severity stratification. Severity is assessed using the rheumatoid factor (RF), with samples labeled as severe if RF≥100 and mild otherwise (Nielsen *et al.* 2012).
- Covid-19 (Chassagnon *et al.* 2021): The dataset of 693 patients affected by Covid-19 reports 543 imaging and clinical variables. The task considered on this dataset is patient severity stratification three days after diagnosis. We compare to algorithm KEA, and physician experts’ performance from Chassagnon *et al.* (2021).
- TCGA (Thorsson *et al.* 2018): The dataset is coming from The Cancer Genome Atlas (TCGA https://www.cancer.gov/tcga) including 20 531 genes for 7045 samples across 24 different tumor types. The task considered on this dataset is cancer types classification using RNAseq gene expression data. References (Battistella *et al.* 2019, Battistella 2021) propose this task to determine a small set of genes characteristic of tumor type that could be used to devise a more time and cost-efficient gene screening method to determine metastasis’ primary sites clinically. They provide a baseline method named COMBING.
- MI (Golovenkin *et al.* 2020): The dataset of Myocardial Infarction (MI) patients includes 1700 samples, and 111 clinical and metabolites. The task considered on this dataset is MI complications after one-day prediction. For conciseness, these results are reported in Supplementary Material.

Each dataset was chosen because of the diverse challenges it presents: a high dimensionality and a complex task with many classes (TCGA), a high dimensionality with a low number of samples and two types of features (RA-MAP), an unbalanced dataset with data extracted from different modalities and centers (Covid-19), and multiple noisy or irrelevant features (MI). In addition, the tasks tackled are well-studied. The TCGA is a referential dataset in genomics (Tomczak *et al.* 2015). Covid-19 has been used in several articles and designed for this specific task (Chassagnon *et al.* 2020, 2021, Battistella *et al.* 2024). RA-MAP and MI are representative benchmarks of usual medical cohorts presenting a limited number of samples, noise, and a high number of features. They both address tasks of prime importance (Nielsen *et al.* 2012, Martin-Gutierrez *et al.* 2022, Farah *et al.* 2022, Oliveira *et al.* 2023). To ensure the robustness of our conclusions, we report results for four different seeds generating four different training and test splits on each dataset.

To evaluate the performance of our proposed approach, we compare our results to several state-of-the-art pipelines (Supplementary Material S0.2):

- Majority Voting (MV) Ensemble Feature Selection (Caba *et al.* 2021) is an ensemble feature selection method that keeps the most frequently selected features across all splits by all composing feature selection techniques.
- Weighted Majority Voting (WMV) Ensemble Feature Selection (Saeys *et al.* 2008) is an adaptation of MV in which we consider the average importance weight given to a feature by the feature selection techniques.
- Tree-based Pipeline Optimization Tool (TPOT) (Le *et al.* 2020) is an AutoML method that relies on a genetic algorithm to explore thousands of possible ML pipelines and exploit the best ones.
- COMBING (Battistella *et al*. 2022) is an unsupervised method, that relies on clustering techniques to identify a relevant set of complementary genes, originally designed to discover cancer biomarkers on TCGA.
- Knowledge-driven Ensemble Approach (KEA) (Chassagnon *et al.* 2021) is a method leveraging the pipeline we adapt in this paper with an MV ensemble feature selection technique fine-tuned to obtain state-of-the-art results on the Covid-19 dataset. It relies on expert radiologists’ knowledge to tune the feature selection by separating the features into medically relevant categories and granting more weight to studied features.
- GHOST (Battistella *et al.* 2024) is a higher-order distance learning approach applied to Covid-19 using conditional random fields to define the best-suited metric for the task.
- No Selection: All the features are used for the prediction as a sanity check of the performance of the feature selection (Not performed on TCGA for tractability issues).
- No Ensemble: The feature selection methods are used alone, and we keep the one with the highest performance. This constitutes a sanity check of the relevance of the ensemble approaches (Not performed on RA-MAP and TCGA for tractability issues).

The MV and WMV baseline methods rely on the same pipeline as the proposed GE except for the ensembling of the feature selection, allowing the testing of the relevance of the proposed graph-based method. Thus, MV and WMV represent ablation studies of k-Heavy and k-W Heavy regarding the use of graph theory, while k-Heavy is an ablation study of k-W Heavy regarding the use of the co-importance weights. As alternative ML pipelines, TPOT, Combing, and KEA allow for assessing the relevance of the proposed ML classification framework.

We also assessed the relevance of the ML-based approaches against two field-specific medical scores:

- Eular Score (ES) (Biliavska *et al.* 2013) is a clinical score designed to characterize the severity of RA in patients.
- Consensus of Physicians (CP) (Chassagnon *et al.* 2021) is a combination through an MV approach of the predictions of three expert radiologists performed on Covid-19, relying on the patients’ imaging and clinical information.

### 2.4 Evaluation

**Figure 2.**
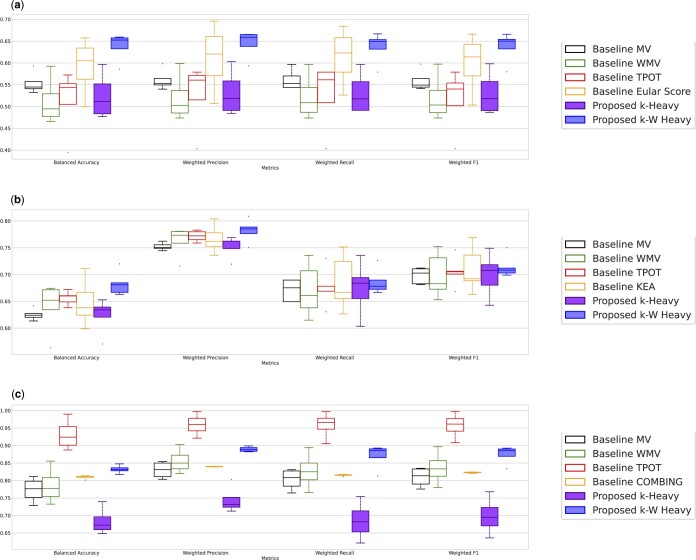
Classification performance on four datasets. We compare the results of the proposed method, k-W Heavy, with several state-of-the-art ensembling feature selection, task-specific, and autoML approaches relying on four different metrics: Balanced Accuracy, Weighted Precision, Weighted Recall, and Weighted F1-Score. Four medical datasets are used, and the results presented are averaged over four different seeds to split the datasets between training and test sets. The proposed k-W Heavy outperforms all the other approaches on all the tasks except TCGA, overall providing higher performance with lower variance. On the TCGA dataset, k-W Heavy presents better results than the feature-selection-based approaches, only outperformed by the autoML approach TPOT, which is not constrained by selecting a low number of features. (a) RA-MAP. (b) Covid-19. (c) TCGA.

To evaluate the performance of each method, we use a range of classification metrics, including Balanced Accuracy (BA), Weighted Precision (WP), Weighted Recall (WR), and Weighted F1 score (WF) on training (Tr) and test (Te) (definitions in Supplementary Material S0.1). The results are presented in boxplots aggregating the results over all seeds for better interpretability in Fig. 2 [see Supplementary Material S1 for the exact numbers with confidence intervals (CI) and Supplementary Material S2 for the performance on each seed to assess their robustness].

#### 2.4.1 Graphical signature evaluation

We rely on network analysis to provide a more biologically-based evaluation of the quality of the signatures of features composed of genes. It has been shown that in the Protein–Protein interaction network (PPI), we can define a disease module Barabási *et al*. (2011) to characterize disease-associated gene interactions. In this article, to define the PPI, we relied on the work from Gysi and Barabasi (2022), which combined and curated 21 datasets to reach 536 965 interactions for 18 217 proteins. In addition, they identified the genes associated with 861 diseases, including RA. We defined the RA module from those genes with their Largest Connected Component (LCC) in the PPI. The LCC is the largest subgraph for which a path exists between any pair of nodes in the subgraph. Then, the evaluation of the genes selected by a given ensemble method is performed regarding two criteria.

- The selected genes have to provide relevant information on the disease. Thus, their distance in the PPI to the disease LCC has to be minimal.
- The selected genes have to provide good coverage of the possible biological processes. Thus, their distance from one another has to be important (Safari-Alighiarloo *et al.* 2014).

Each criterion by itself brings relevant information. However, it is their association that ensures a good description of the relevant area of the PPI around the disease module. We defined two graphical metrics to evaluate a feature selection method according to those principles. The first metric is the average distance in the PPI of a selected gene to the LCC of the disease of interest. The second metric is the average distance between two selected genes. Those metrics are then averaged over the different seeds considered in the experiments. Besides, we defined a random signature of 100 genes selected without repetition per considered seed to obtain referential measures.

#### 2.4.2 Genome wide association study analysis

Genome wide association study (GWAS) (Welter *et al.* 2013) is commonly used to identify genomic variants statistically associated with a risk for a disease or particular trait. Toward a better interpretation of the genes selected and a quantitative comparison between the different selection methods, we assumed that the coverage by GWAS of the genes associated with RA is good enough to be used as a referential. We considered the study EFO_0000685 of the GWAS catalog to characterize RA. We estimated the overlap between the selected genes and GWAS ones for each selection method, and its statistical significance estimated through a hypergeometrical test. More specifically, we defined the hypergeometrical test as the probability:

(1) $$p(k,M,n,N)=\frac{\left(\begin{array}{c} n\\ k \end{array}\right)\left(\begin{array}{c} M-n\\ N-k \end{array}\right)}{\left(\begin{array}{c} M\\ N \end{array}\right)}.$$

In our settings, *k* represents the size of the overlap between the selected genes and the GWAS, *M* is the total number of genes in the PPI, *n* is the number of coding genes associated with RA in GWAS, and *N* is the total number of selected genes. Finally, we estimate the probability of randomly obtaining a larger overlap using the *P*-value that corresponds to 1−cdf(p(x,M,n,N)) where cdf is the cumulative distribution function.

## 3 Results

### 3.1 RA-MAP

The RA-MAP dataset presents a significant challenge for ML due to its high dimensionality and small sample size. This challenge is obvious in Fig. 2a, highlighting that none of the baselines reach 60% average performance on any metric. The best ML baseline, MV, reports 55% of average BA and 56% of average WP, WR, and WF on test (Table 2), which is indistinguishable from a random outcome.

**Table 2.** Average distance in the PPI network between the genes selected by the different approaches on different seeds (Intra-Signature) and between the selected genes and RA disease LLC (LCC).

	Intra-Signature	LCC
Random	1.87 (+0%)	1.52 (+0%)
MV	2.28 (+21.65%)	1.55 (+1.97%)
WMV	2.22 (+18.86%)	1.53 (+0.33%)
k-Heavy	2.22 (+18.80%)	1.47 (−3.42%)
**k-W Heavy**	**2.44 (+30.62%)**	**1.43 (**−**5.99%)**

The k-W Heavy reports a higher Intra-Signature than the baselines ensuring that the genes selected account for different biological processes. At the same time, k-W Heavy reports a lower LCC, which proves the biological relevance of the selected genes for RA.

The bold values show the best performing method.

In contrast, the proposed k-W Heavy method successfully extracts signal from the data, achieving 64% BA, WP, WR, and WF. Moreover, the maximal difference between the metrics on the training and test sets for MV is 30%, while the proposed method reports a reduced difference of only 11%, indicating its ability to avoid overfitting and generalize better. Most importantly, MV requires 21 features on average to perform the classification, while the k-W Heavy uses only 9.5 features. The improved performance of the proposed K-W Heavy over MV and WMV proves the relevance of graph-based approaches for extracting a signal in complex medical datasets. Besides, only k-W Heavy outperforms the use of classifiers without any feature selection (see Supplementary Table S3). At the same time, the improved performance over TPOT highlights the need for feature selection and ensemble techniques to improve robustness and prevent overfitting.

Finally, we find that k-W Heavy performs better than the Eular Score, a clinical score used in patient care, and improves BA by 5%, WP and WR by 3%, and WF by 4% while presenting better training performance by 22% on all metrics.

Next, we assess how the genes selected by k-W Heavy relate to biological processes associated with RA. For this purpose, we constructed the human PPI, whose 18 217 nodes are proteins, and the 536 965 links are the experimentally detected binding interactions between proteins derived from 21 public databases (Gysi and Barabasi 2022). We labeled the 27 genes uniquely selected over the 4 different seeds on the PPI and computed the average pairwise distance between the signature proteins (intra-signature distance). We assume that the distance between proteins in the PPI reflects the difference in the biological mechanisms the genes are involved in, a low intra-signature distance indicating a potential involvement in similar cellular processes and disease mechanisms (Safari-Alighiarloo *et al.* 2014). We also measured the average distance between the signature genes and the 181 genes of the LCC of a list of 391 RA genes collected from the literature (Gysi and Barabasi 2022) (see Section 2 for details). This distance characterizes the closeness of the selected genes to biologically relevant RA genes. We used 400 randomly selected genes to estimate the average distances for random genes.

In Table 2, we report the distances computed for the signatures of the baselines and the proposed GE. We observe that the best baseline, MV, reports an intra-signature distance that is 22% greater than the random distance, while the proposed k-W Heavy has a distance 31% higher than random. This indicates that the proposed k-W Heavy approach selects more diversified signatures, accounting for different biological mechanisms with less redundancy. We illustrate this property in Supplementary Fig. S3, where we observed long paths between the selected genes, highlighting the difference in biological processes. Besides, the average distance to the LCC of the best baseline, WMV, is higher than the random distance by 0.33%. On the other hand, k-W Heavy reports an average distance to the LCC that is 6% lower than random. Therefore, the genes identified by the k-W Heavy method are closer to the known RA genes than the baseline ones.

Finally, using the curated collection of genome-wide associations GWAS (Welter *et al.* 2013), we identify genes statistically associated with RA (see Section 2). We find that the baselines had respectively selected 2 (MV) and 3 (WMV) genes associated with a known increased risk for RA, which amounts to the non-significant p-values of 0.298 and 0.0582 when taking into consideration the total number of features selected. In contrast, with only 27 selected genes and 3 genes associated with an increased risk for RA, namely FBXL19, SFTPD, and TPT1, k-W Heavy provides a statistically significant overlap with known RA disease genes (*P*-value = .0449). This demonstrates the ability of graph-theory-based methods for feature selection ensembling to identify genes with greater biological relevance.

In summary, the proposed K-W Heavy extracted a more compact signature of biologically significant genes, which empowers improved classification performance over ML-based as well as clinical baselines.

### 3.2 Covid-19

For the Covid-19 dataset, the best baseline, TPOT, reports 66% average BA, 77% WP, 68% WR, and 70% WF. The proposed k-W Heavy achieves a 68% BA, 78% WP, 69% WR, and 71% WF, a better performance associated with a lower amplitude in all the other metrics (Fig. 2b), a proof of greater robustness. We report in Supplementary Table S4 the performance of a baseline using a single feature selector without consensus. This sanity check shows that only the k-W Heavy ensemble technique offers improved performance over the absence of ensemble technique. In addition, on the data split KEA was designed for, a center-wise split using samples from unseen hospitals on the test (See Supplementary Table S5), k-W Heavy achieves 72% BA, outperforming by 2% KEA, and by 5% a consensus of three expert physicians. In this case, k-W Heavy is the only ensemble technique outperforming the baseline without any feature selection. Furthermore, k-W Heavy even outperforms GHOST (BA of 71%), a computationally expensive feature selection method that identifies higher-order relations. This demonstrates that GE offers improved performances compared to approaches leveraging complex interaction or combining data and knowledge-driven information, which require field experts to help handpick the features. Additionally, the performance variance over the different seeds is largely reduced compared to KEA (Fig. 2b), indicating that relying on a knowledge-driven approach induces a bias. The CI width is smaller for k-W Heavy than KEA, at 6% versus 11%.

We provide in Supplementary Material S0.5 the runtime of Graphical Ensemble feature selection for the considered numbers of features, demonstrating that our approach is tractable.

Note that the observed superior performance of k-W Heavy was obtained with 9.4 signature genes on average, while the best baseline (WMV) needs 16. GE enables us to combine imaging and clinical features synergistically to improve the results over Combing, which was shown to fail to use clinical variables along with imaging ones.

In summary, the proposed K-W Heavy was able to identify a more compact signature allowing for better results and a lesser variance beating all the baselines, including a consensus of physicians. We cannot provide a network-based analysis on the Covid-19 biomarkers as the considered features are not genes and thus cannot be mapped to the PPI.

### 3.3 Cancer

For the TCGA dataset, we find that all methods offer results above 80% BA, with the AutoML pipeline TPOT outperforming the proposed k-W Heavy. The better performance of TPOT on this dataset is likely due to our choice to limit our pipeline to a small number of genes, which appears to be insufficient for leveraging the full potential of the proposed pipeline on this task. Indeed, in Battistella (2021), the authors show that almost perfect performances are reached by selecting 100 random genes, illustrating that the relevance of the task dwells in minimizing the number of genes while maintaining high performance. We reproduced the experiment and obtained 91% BA, 93% WP, 92% WR, and 93% WF. We limited the number of features to demonstrate the relevance of GE in obtaining better explainability, as well as to improve our understanding of the biological mechanisms by limiting our attention to fewer biomarkers. Indeed, as highlighted in Battistella (2021), to design a better gene screening method for tumor metastasis, we must rely on a small number of selected genes is primordial as it enables a better understanding of their potential connection to the tumor type. As we discuss in Supplementary Material S3, the maximal number of genes considered does not allow to reach a plateau in performance. While TPOT reports the best overall performance, the proposed k-W Heavy provides increased performance compared to the ensemble feature selection baselines by 4% BA and WR, and 3% WP and WF. Besides, k-W Heavy presents more reliable results with a CI width in BA of only 3% against 10% for TPOT. In addition, note that the signature of the identified biomarkers enables us to compete with the results obtained with COMBING’s signature, which contains half more genes. This state-of-the-art method was proven to identify genes well-suited for tumor characterization in Battistella (2021). Moreover, COMBING has been defined with a part of our test set, which entails a high risk of data leakage. This happens when test samples are used in the learning phase, and causes an undue boost of COMBING signature’s performance that would not generalize to other datasets.

In summary, the proposed K-W Heavy approach provides better, more stable results than the ensemble feature selection baselines. Unlike TPOT, it produces interpretable results thanks to selecting a small signature of genes and offers enhanced performance when used to select a larger signature.

### 3.4 Generalization

The proposed GE offers additional opportunities for generalization to improve the accuracy and interpretability of ML. Further considerations about its application to medicine are discussed in Supplementary Material S0.11. First, the weight matrix used to build the graph from which the k-heaviest subgraph is extracted can be enriched with diverse information types. This allows us to include any notion of similarity between features derived from biological properties, expert knowledge, information extracted from the literature, and graphical models. For example, incorporating information extracted from network analysis can provide biological and structural insights for the feature selection task. An interesting possibility would be to consider the network-based distance between the genes and the disease module (Barabási et al. 2011) for an additional weighting of the co-importance graph. It is also possible to consider any notion of penalization on feature pairs, such as the acquisition cost of obtaining features from different modalities, to favor signatures from the same modality.

In addition, the concept of a graph can be extended to a hypergraph, allowing us to consider patterns emerging in the co-selection graph. For instance, triplets of nodes that have been of interest for medical applications (Benson *et al.* 2016) could be investigated to model feature interactions.

Also, GE can consider any cost over combinations of features. For example, if causality information on the features is available, the selection of confounders could be penalized. In this case, the identified signature will be a trade-off between the informativeness and lack of redundancy ensured by feature selection techniques and the added properties. Finally, the proposed approach can be used in a regression context by modifying the aggregated models.

## 4 Discussion

In conclusion, we proposed a novel class of ensemble feature selection techniques relying on graph approaches called GE. We tested the proposed technique’s relevance for various medical tasks with a fair and thorough comparison to state-of-the-art data-specific methods.

We extensively explored the set of hyperparameters of the baseline methods to ensure that we cover a significant portion of the search space. Hence, we avoid the possibility of selecting parameters working exclusively with our method. We compared 8 feature selection methods and 15 classifiers, each of the 4 ensemble feature selection methods was used with 4000 different hyperparameters, and the classifiers tuning took 130 000 cross-validation. We found that the proposed approach selects more stable features over different experimental conditions and enables more robust classification results.

We performed different experiments to identify the context in which GE offers superior performance. First, as demonstrated with the RA-MAP task, GE can better extract signal from noisy features, making it a reliable choice on challenging datasets. Second, by offering a signature geneset smaller in size on all experiments, with less redundancy and more biological relevance, as demonstrated by the network analysis with the RA genes module, GE can identify biomarkers for a disease that would be less expensive and time-consuming to use in routine treatment. Third, GE performance presents a lower variance, implying a better generalization ability to new datasets. Finally, k-W Heavy provides a graphical overview of the relation between the features and their complementarity. Also, note that GE has been mainly studied in this manuscript as a method to select a small number of features as a biomarker. Indeed, when trying to find the minimal set of predictive features, the elimination of redundant and non-predictive features is all the more important. For tractability’s sake, we recommend using the approximate version of the algorithm when selecting a larger number of features.

GE offers the potential to integrate network-based analysis into the weight matrix, enriching the ensemble feature selection with more complex biological interaction information. These adaptable approaches allow the incorporation of expert knowledge, biological information, or even literature-derived biomarkers directly into the feature selection process. We can also generalize the study of the dependency on the training set size we performed on Myocardial Infarction, examining the robustness of GE to different sample sizes.

Graphical models have demonstrated their value in many fields, including deep learning, and here, we show their utility for ensembling methods. We introduce graph-theory-based ensemble feature selection techniques whose performance has been demonstrated on four different medical tasks. From a more general perspective, this work offers unique insights into how to combine graph theory and ML, demonstrating the usefulness of graph structures for ensemble feature selection.

## Supplementary Material

btae341_Supplementary_Data

## Data Availability

The data used in this article is publicly available. RA-MAP dataset was published on Gene Expression Omnibus in 2017 by John C, Ehrenstein M, Barnes MR, Lendrem D, and Isaacs JD with the identifier GSE97810. Covid-19 dataset is available at https://github.com/ebattistella/Covid-MedIA. MI dataset is available at https://archive.ics.uci.edu/ml/datasets/Myocardial+infarction+complications. TCGA dataset is available at https://portal.gdc.cancer.gov/ and was downloaded on 21 September 2021.

## Code availability

The code used in this article is available as modular functions that automate feature selection, classification, and clustering while allowing for the comparison of several state-of-the-art methods at https://github.com/ebattistella/auto_machine_learning.
