# Supplementary material for: Improving the performance and interpretability on medical datasets using graphical ensemble feature selection
Source: Bioinformatics. 2024 Jun 5;40(6):btae341. doi: 10.1093/bioinformatics/btae341 (PMC11187494; doi:10.1093/bioinformatics/btae341)
Supplement: btae341_Supplementary_Data [file btae341_supplementary_data.pdf]

Supplementary Material

S0.1 Assessment Metrics

In this section, we describe the different assessment criteria we resorted to. First, we detail the metrics used to evaluate the classification results of the proposed pipeline. Then, we describe the method to evaluate the relevance of the signature of features.

Regarding the classification metrics, we relied on criteria allowing multi-class classification with unbalanced classes as Balanced Accuracy (BA), Weighted Precision (WP), Weighted Recall (WR), and Weighted F1-score (WF). In a general multi-class setting for a set of classes C, we consider each class $c \in C$ in one-versus-rest. We denote the set of true positives of class c as y_c, and the set of samples predicted positive for class c as y_c^P. We define the precision (P), recall (R), and F1-score (F) of a class $c \in C$ against all the other classes as:

$$P(c) = \frac{\mid y_c^P \cap y_c \mid}{\mid y_c^P \mid},\ R(c) = \frac{\mid y_c^P \cap y_c \mid}{\mid y_c \mid},\ F(c) = \frac{2P(c)R(C)}{2P(c) + R(c)}$$

Recall corresponds to the True Positive Rate for the class c. Precision is the Positive Predictive Value. F1-score represents the harmonic mean between R and P. Then, the weighted version WS of a metric $S \in \{P, R, F\}$ corresponds to the average of S over all classes of C weighted by the size of the classes:

$$WS = \frac{1}{\sum_{c \in C} \mid y_c \mid} \sum_{c \in C} \mid y_c \mid S(c)$$

Finally, the balanced accuracy is the proportion of correctly predicted samples normalized by the number of samples in the class. Formally, it is defined as:

$$BA = \frac{1}{\sum_{c \in C} \mid y_c \mid} \sum_{c \in C} \frac{\mid y_c^P \cap y_c \mid}{\mid y_c \mid}$$

To ensure the robustness of the results, we considered several seeds for splitting the data. We compare the results and signatures over those different seeds through averaging and confidence intervals. We considered the following definition of Confidence Interval (CI) to assess the results' uncertainty.

Non-parametric CI: require an empirical score distribution (EMS) defined here by our set of experiments. In this case, for a significance level of α:

$$\begin{aligned} Upperbound =& percentile(EMS, 100 - \alpha/2) \\ Lowerbound =& percentile(EMS, \alpha/2) \end{aligned}$$

with $percentile(D, n)$ the $n - th$ percentile of the distribution D.

S0.2 Baselines

To assess the performance of the proposed approach, we compare our results to different standard and state-of-the-art pipelines. In particular, we evaluated the proposed ensemble feature selection technique relevance by comparing two standard methods leveraged by our proposed pipeline. Also, we assessed the relevance of the proposed framework compared to a state-of-the-art AutoML method. Finally, we analyzed our results in contrast with the best-published results obtained on these

tasks when available. More specifically:

- Majority Voting (MV) Ensemble Feature Selection [1]: This elementary ensemble feature selection method is used in our pipeline instead of Graphical Ensembling. It consists in keeping the features selected the most over all splits by all the feature selection techniques. This simple method is agnostic of the features selected together by a same feature selection model. This ensemble feature selection technique requires defining thresholds on the number of features selected for each of its composing methods. Those thresholds were optimized manually to obtain a similar number of features selected by each technique. It is the counterpart of proposed k-Heavy method.

- Weighted Majority Voting (WMV) Ensemble Feature Selection [2]: This method is an adaptation of MV in which we consider the average importance weight given to a feature by the feature selection techniques. It provides a more nuanced assessment than MV and dispenses with the need to define eventual selection thresholds for the feature selection techniques. It is the counterpart of proposed k-W Heavy approach.

- Tree-based Pipeline Optimization Tool (TPOT) [3]: This AutoML method relies on a strongly typed genetic algorithm to explore thousands of possible machine learning pipelines and exploit the best ones. It has been specifically designed to leverage biomedical data.

- COMBING [4]: This unsupervised method relies on clustering techniques to identify a relevant set of complementary genes. It has been designed explicitly for discovering cancer biomarkers on the TCGA dataset. Thanks to the defined

signature, state-of-the-art results have been obtained on cancer type classification. This signature was obtained on a subset of the TCGA. To enable the comparison, we used their signature with the proposed classification pipeline.

- Eular Score (ES) [5]: This clinical score has been experimentally designed to characterize RA patients' status. It relies on the number of swollen joints of the patient and is commonly used in daily practice.

- Knowledge-driven Ensemble Approach (KEA) [6]: This method leverages the pipeline we propose to adapt in this paper with an MV ensemble feature selection technique. Moreover, it relied on expert radiologists' knowledge to better tune the feature selection by separating the features into medically relevant categories and granting more weight to known features of interest. It has been fine-tuned to obtain state-of-the-art results on the Covid-19 dataset.

- Consensus of physicians (CP) [6]: In this article, three expert radiologists performed the same classification task on the Covid-19 dataset. They relied on the patients' imaging and clinical information, and their predictions were combined through an MV approach.

- GHOST [7]: This higher-order distance learning approach relies on conditional random fields to define the best-suited metric for a given classification task. In particular, it has been successfully applied to the Covid-19 dataset.

Those baselines aim to establish that the ensemble method proposed outperforms the most commonly used ones on the same proposed AutoML pipeline, that the proposed AutoML pipeline is a relevant proposition outperforming a state-of-the-art AutoML

technique, and that our results are comparable or improved relative to the cutting-edge methods tailored to the specifics tasks at hand.

S0.3 Model Selection Rules

We formalize an original rule scheme to select the best model r^* among a set of models $\mathcal{R}$ trained over k-fold cross-validation. We rely on a set of evaluation metrics $\mathcal{M}$ defined in Supplementary Material S0.1, a set of threshold pairs $\mathcal{T}$, and a set of functions $\mathcal{F}$. A metric assessment $m(r)$ for $m \in \mathcal{M}$ is a pair of evaluations of a model $r \in \mathcal{R}$ on training and validation, denoted as $m(r) = (m^{Tr}(r), m^{Val}(r))$. By assessment on training (respectively validation), we mean the average of the corresponding metric over the training (respectively validation) sets of the cross-validation. A selection rule is then defined by a set of p indexes quadruplets $\mathcal{S} = \{(m_i, t_i^{min}, t_i^{max}, f_i)\}_{\{i \in [1,p]\}}$ such as $\forall i \in [1, p]$, $m_i \in M$, $(t_i^{min}, t_i^{max}) \in T$, $f_i \in F$, and:

$$r^* = R_{b*} with\ b^* = \arg\max(\{\sum_{i \in [1,p]} m_i^{Tr}(r),\ r \in \mathcal{R},\ \forall i \in [1, p], t_i^{min} \leq f_i(m_i(r)) \leq t_i^{max}\})$$

This means that the best model is selected as the model presenting the highest assessment score under the constraints enforced by the rule. The assessment score is defined by the sum of the metric assessments on validation. A constraint $i \in [1, p]$ of the rule imposes the image of $m_i(r)$ by f_i to be between the thresholds t_i^{min} and t_i^{max}.

The rule we used is $S^* = \{(BA, 0, 0.05, -), (WP, 0, 0.05, -)\}$ where $-(BA(r)) = BA^{Tr}(r) - BA^{Val}(r)$. The rule amounts to select the model with the highest sum average BA and WP on validation under the constraint that the discrepancy between the average BA and WP on training and validation is no more than 0.05 and that

BA and WP are superior on training than on validation. More details are provided in Supplementary Material S0.4.

S0.4 AutoML Adaptation

Reproducibility and fairness are critical issues when evaluating proposed approaches against gold standards [8]. These issues are generally related to the difficulty of fine-tuning all the different baselines and the proposed method equitably. This is particularly challenging when considering feature selection techniques, as they add a layer of hyperparameters to the process. AutoML offers a promising solution by providing automated frameworks for machine learning that do not need any manual fine-tuning of the parameters to define and train a model, defined as a classifier ready to predict after having been trained on a dataset with specific features and parameters [9]. Notwithstanding, AutoML techniques' efficiency relies on the criteria used to identify the best model [10] while the most straightforward approach of relying on validation performance only favors overfitting [11]. Often, AutoML refers to deep learning architectures. However, neural networks require considerable amounts of data and lack interpretability [12]. As a result, we will prefer conventional machine learning methods.

The framework introduced in [6] we propose to adapt, even though robust and promising, is an unwieldy and complex to tune architecture. We propose a very straightforward process to tune the numerous hyperparameters it possesses and tackle the model selection problem. To perform the hyperparameters tuning for both the classifiers used at the feature selection step and the classification step, we chose a random search with the maximization of the BA as the target. Then, using the

ensemble method detailed in Subsection 2.1.1, we extract a signature of features. Finally, to determine the best model, we rely on the model selection rule defined as $S^* = \{(BA, 0, 0.05, -), (WP, 0, 0.05, -)\}$. The following criteria summarize it:

i Maximal sum of average BA and average WP on validation.

ii Discrepancies between average BA and WP on training and validation < 0.05.

iii Average BA and WP on training superior to the validation ones.

So, we aimed to select the model offering the best performance (i) while avoiding the pitfall of overfitting (ii) or underfitting (iii). In this study, the choice of retaining the combination of balanced accuracy and weighted precision is motivated by the benefit of using multiple metrics to avoid overfitting or degenerate results. Also, balanced accuracy is a standard assessment metric for unbalanced datasets. While weighted precision offers clinical relevance by allowing us to characterize the number of positive samples we are missing. Indeed, in many medical tasks, we want to ensure that patients presenting a disease are correctly diagnosed and treated. In this case, false negatives are the critical parameter [6]. We also analyze the impact of different rule choices. The model selection rule can be adapted according to the task at hand, which can be easily implemented using the code we provide. In the Supplementary Material Section S4, we study the influence of the chosen rule. In addition to the previously defined metrics, statistical tests for model selection could also be considered as the Bayesian Information Criterion (BIC), the Akaike Information Criterion (AIC), or the Minimum Length Description (MDL).

S0.5 Runtime Analysis

The feature selection takes 13.5 minutes in total.

# Features	2	5	10	15	20	25	26	27	28	29	30
Time (min)	$7e^{-3}$	$4e^{-3}$	$4e^{-3}$	$4e^{-3}$	$5e^{-3}$	$4e^{-2}$	0.13	0.56	2.6	9.9	36

Table S1: Runtime analysis of k-W Heavy on the Covid-19 dataset for the considered values of k.

# Features	10	20	30	40	50	60	70	80	90	100
Time (Seconds)	0.19	0.15	0.17	0.15	0.17	0.14	0.20	0.20	0.22	0.27

Table S2: Runtime analysis of the approximate k-W Heavy with factor 2 on the Covid-19 dataset.

S0.6 Myocardial Infarction

For MI, the best baseline, MV, reports 68% average BA, 70% WP, 66% WR, and 66% WF. The k-W Heavy presents the best results with 69% BA, 71% WP, 67% WR, and 67% WF, and a reduced variance across the seeds (Figure S6a). Besides, it is interesting to notice that its counterpart without relying on graph theory, WMV, presents performances equivalent to the approach without feature selection and ensembling.

The large sample size of the MI dataset (1,700 samples) allowed us to study the influence of the training set sample size on the predictive power of the proposed approach. We considered subsets of the training data as defined in Methods S0.7 to quantify the influence of the number of samples on the classification performance and the density of the subgraph selected by k-W Heavy. We observe an inflection point leading to a plateau at roughly 600 training samples (50% of the total training samples available) in both the classification performance and the density (see Figure S8), indicating that around 600 samples are sufficient to achieve optimal results. Therefore,

this correlation between the impact of sample size on density and performance enables the use of density to determine the number of samples needed for biomarker discovery. Besides, it would be computationally valuable to extend the use of density to determine the number of features to select. In this case, the classification would not need to be performed and compared over the different numbers of features (further results are reported in Supplementary Materials S5).

In summary, for MI, Graphical Ensembling reports results comparable to the baselines while offering the computational advantage of relying on density considerations to select the best feature signature.

S0.7 Sample Size Influence

We studied the influence of the number of samples on a classification pipeline performance and the selection of features. We carried out the experiments on the MI dataset as it is the only one with enough samples per class to perform a meaningful study. Also, an interesting aspect of the sample size's influence is examining the risk of considering a biased test set. In this case, a model's good performance would not guarantee any generalization property. Toward this end, we considered two distinct scenarios. The first one models the aforementioned issue with small data sets by considering a subset of the patients and performing the split between train and test sets on this subset. The second scenario illustrates the effect of sample size on the definition of a good model. There, we take a unique test on the whole dataset and then consider subsets of the train set. The fact of keeping a single sizable test set allows us to perform a better, unbiased evaluation of a model's results when the former scenario outlines the inherent bias of a small test set.

We report the average classification performance of the best models over the seeds for each sample size, along with the associated density of the selected features in the co-selection graph. This way, we ensure we have enough samples to reach a plateau in performance and correlate this plateau with the evolution of the density.

S0.8 k-Heavy Performance Analysis

k-Heavy is underperforming MV on the RA-MAP and TCGA tasks. This can be explained by the very large number of genes involved (over 20,000) and the high redundancy between genes. Because of that, it is very likely that the same genes would only be selected together sometimes, causing the graph to be very sparse and potentially spurious as the amount of noise can be high. In that case, the k-Heavy subgraph will be ill-defined, with an unstable change of density when increasing the number of features. Indeed, we present in Figure S1 the change in density in percent when increasing k. A higher number of selections would likely solve this issue, but is expensive to obtain, as fitting feature selection methods on so many features is time-consuming. Even so, k-W Heavy does not face this issue as the information brought by the importance score provides a complete graph, thus much more robust information. In this case, the structure of a feature module is again well-defined. This is supported by the superior results of k-Heavy compared to MV on the Covid-19 and MI tasks, which present smaller feature spaces and the dominating performances of k-W Heavy compared to all the other feature selection methods.

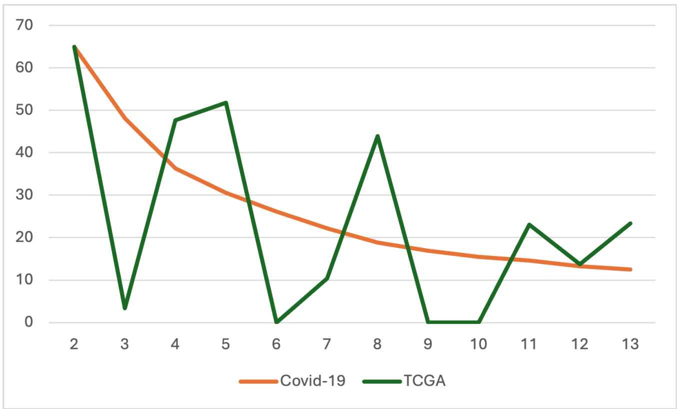

Figure S1: Density Evolution Comparison. Density changes in percent when increasing the number of features in the k-heavy subgraph.

S0.9 Definition of the Density

An alternative possibility to identify the subset of features S the most relevant in the co-selection graph would have been to consider the usual notion of densest subgraph, i.e., a subgraph maximizing the edge density. However, it has been empirically observed that this definition tends to generate sizable subgraphs [13], which is not appropriate for our objective of feature selection. We performed experiments to assess the usability of this technique and support this conclusion. Generally, the maximal densest subgraph, generated using the linear programming algorithm proposed in [14], includes hundreds of nodes and fails to achieve our objective of overfitting mitigation. For instance, an average of 230 genes were selected using this method on the RA-MAP dataset. Therefore, we abandoned the notion of densest subgraph in favor of the better-suited k-Heaviest Subgraph.

S0.10 Filtering

It is common practice to perform a pre-filtering of the features according to known relevant biomarkers, pathways, or simply correlation to the objective. However, experiments on the RA-MAP dataset using correlations and/or biological information for a pre-filtering purpose show they are detrimental to the performances. More specifically, we tried to consider only RA immune-system-related genes and an ANOVA f-value test filtering. In all the cases tested, the results significantly decreased to random ones. Thus, we did not apply the whole comparison pipeline to these settings for computational tractability issues. This highlights that the associations between features at stake are complex and multivariate. Therefore, it is beneficial to directly integrate correlation and biological information in the ensemble feature selection. Indeed, experiments performed without the feature selection techniques derived from statistical tests underperformed. It highlights the relevance of those statistics to select features better correlated to the outcome and eliminate noise.

Yet, another important difficulty out of the scope of this study is the choice of the feature selection components used in the ensemble method. If one feature selection technique consistently chooses the same set of irrelevant features, it will significantly impair the consensus signature, whatever the ensemble technique used. So, identifying which selection models are relevant might have a massive impact. This point does not affect the validity of the comparisons reported in this paper, as all the aggregation methods will be impacted similarly.

One possible solution to this issue would be to evaluate the relevance of the features selected by each component through their correlation to the outcome or classification performance and discard the ones below a given threshold. A parameter-free possibility

enabled by the proposed k-W Heavy approach is to weigh the features' importance by the correlation or the classification performance of the feature selection technique.

S0.11 AutoML for Medical Applications

Due to the vast number of experiments at stake in this article, only a systematic approach could have ensured a fair assessment of the proposed method's superiority over the baselines under the same comparison. Besides, from a fairness perspective, this pipeline had to leverage several classifiers of very different properties to capture all possible types of correlations in each signature of features.

While Machine learning presents limitations and risks when applied to the medical field, AutoML techniques provide a tailored-to-the-task machine learning framework usable by non-experts. Notwithstanding, strong statistical expertise is required to design and model the task and analyze the model results, especially when tackling medical tasks, sensitive by nature. To facilitate this work, we estimate confidence intervals, indicate the different features' number of selections for better interpretability, and automatically perform the preprocessing steps to guarantee the absence of data leakage.

Additionally, it is important to note that machine learning techniques aim to identify variables that are correlated with the objective, but this does not necessarily imply causality. The identified features even strongly correlated to the outcome and powerful predictors for the task might not have any direct biological relation with the disease.

Moreover, while the automatic pipeline proposed in this work exhibits excellent performance, manual fine-tuning is likely to improve the results significantly. Nevertheless,

the pipeline provides a strong baseline for hyperparameter optimization.

S0.12 Extent of the Experiments

It is relevant to highlight the exceptional number of experiments performed for this study. First, the number of ensemble selections performed is already over $4,000$ for each ensemble method, so over $16,000$ in total. Second, the thresholds their components rely on must be manually fine-tuned on a case-by-case basis for each task. We tuned the non-weighted feature selection technique thresholds to select a similar number of features using around 200 cross-validations. Third, for tuning the classifiers on each generated signature, we needed over $130,000$ cross-validation. To account for the models' training, we have to multiply these numbers by the number of folds in the cross-validation 10, the number of feature selection methods 8, and the number of classifiers 15. We have to add the experiments using the signatures from the baselines 64 and the AutoML baseline 440, plus several experiments using different settings like the density and filtering techniques discussed in Sections S0.9 and S0.10.

Finally, for the sample size influence on the MI dataset, we have to perform the experiments on each of the 11 other sample sizes.

S0.13 Implementation Details

We implemented all the code in Python 3.7 using the scikit-learn 0.24.1 package, scipy 1.6.2, and TPOT 0.11.7.

Regarding the feature selection, we leveraged the Decision Tree (DT), the Support Vector Machine with linear kernel (linear SVM), the Gradient Boosting (GdBoost) and the AdaBoost (AB) classifiers, the least absolute shrinkage, and the selection

operator (Lasso) method, the chi-square measure (chi2), the ANOVA F-value (F-val) and the Mutual Information (MI). Regarding the classification, we relied on the Logistic Regression (LR), the Stochastic Gradient Descent (SGD), the linear SVM, the polynomial SVM, the radial basis function (RBF) SVM, the sigmoid SVM, the K-Nearest Neighbors (KNN), the Gaussian Process (GP), the Gaussian Naive Bayes (GNB), the Multinomial Gaussian Naive Bayes (MGNB), the DT, the AB, the Random Forest (RF), the Bagging (Bag), and the GdBoost classifiers.

We performed a random search for the classifiers tuning. The parameters to be tuned and the search spaces are the following ones. For LR, the penalty was either l1, l2, or elasticnet, the C followed a uniform distribution between 0 and 50, the solver was saga, the maximum number of iterations was 10000, and the l1 ratio was 0.5. For SGD, the loss was either hinge, log, modified huber, squared hinge, perceptron, huber, epsilon insensitive or squared epsilon insensitive, the penalty was either l2, l1, or elasticnet, the alpha and the l1 ratio followed uniform distributions between 0 and 1, the SVM methods had C parameters following uniform distributions between 0 and 50, the polynomial SVM was used with a degree between 1 and 8, KNN relied on 2 to 50 neighbors and either uniform or distance-based weights. GP leveraged 0 to 10 optimizer restarts, GNB had a smoothing variable following a truncated normal distribution between 0 and 1, of location $1e^{-9}$ and scale $1e^{-3}$, MGNB followed a uniform distribution between 0.01 and 10, DT had a maximal depth between 2 and 30 and a minimal number of samples per split between 2 and 10, AB had between 2 and 100 estimators and a learning rate following a uniform distribution between 0 and 1, RF had between 2 and 200 estimators with maximal depths between 2 and 10, Bag had between 2 and 100 estimators, GdBoost had between 2 and 100 classifiers, a

learning rate following a uniform distribution between 0 and 1 and a maximal depth between 2 and 10. We considered the Lasso method with iterative fitting along a regularization path of length $5e^{-3}$, 400 alphas along the path, and a maximum number of iterations of 1000. We used all the classifiers allowing us to leverage class weights with balanced weights to circumvent the effect of eventual unbalanced datasets. We kept unreported hyperparameters with a default value.

Regarding the ensemble feature selection techniques requiring a selection threshold we used, the order being DT, linear SVM, GdBoost, AB, Lasso, chi2, F-val, and MI, on TCGA and RA-MAP 0.1, $5 \times median$, $2 \times mean$, $10 \times mean$,2, 0.7, 0.7, 0.7, on Covid-19 0.1, $3 \times median$, $2 \times mean$, $0.3 \times mean$,$1e^{-6} \times mean$, 4, 4, 4, and on MI 0.1, $1 \times median$, $1 \times mean$, $0.001 \times mean$,$1e^{-4} \times mean$, 30, 30, 30. We chose those thresholds to obtain similar features for every method and between 60 and 100 features per split and selector.

We experimented with random search optimizations with different numbers of iterations. For the feature selection task, it was in $\{1, 5, 10, 15, 20, 50, 100\}$ and in $\{1, 10, 20, 50, 100, 200, 500, 1000\}$ for the classification. As we do not want an overfitting of the feature selection models, we keep the number of iterations of their optimization scheme limited and below the classifiers' one. For the different steps, we relied on 10-fold cross-validations. Besides, we have compared the results when selecting 2 to 30 features. We averaged the results using 4 different random seeds $\{10, 82, 94, 118\}$ to ensure robustness. Besides, for the Covid-19 dataset, we considered an additional split of the dataset corresponding to the one considered for its original publication in [6]. In this split, the testing set is defined by taking all the samples of 3 different centers. It will be referred to by the seed 0 and called center-wise split.

We experimented with the TPOT framework with the different seeds, population size in $\{1, 10, 20, 50\}$, and a number of cross-validation in $\{5, 10, 20, 50, 100\}$.

Regarding the RA-MAP dataset, we considered all the clinical features that were not directly involved in the computation of clinical scores used to assess severity, like Eular Score. Namely, we considered basophils count, eosinophils count, gdl, gl, hb, lymphocytes count, monocytes count, neutrophils count, plt, wbs, age, height, weight, racial information, sex, and the time since onset of the disease.

In the case of the sample size influence determination, our experiments were performed using subsets of 3%, 5%, 10%, 15%, 20%, 25%, 50%, 75%, 80%, 90%, 95% or 100%. For the $SSID$ scenario, we considered the same four seeds as in the main experiments with their corresponding test sets. Then, for both scenarios, we considered the 10 following seeds $\{1, 11, 21, 31, 41, 51, 61, 71, 81, 91, 101\}$ to split the remaining data.

S0.14 Selected Biomarkers

We report in this section all the features selected by the proposed k-W Heavy approach over the different datasets and seeds.

In RA-MAP:

- Seed 10: C3orf14, SFTPD, FBXL19, PLIN2, PDK4, ZDHHC4, MAP10, ITIH5, LRRC6.
- Seed 82: KDM2B, SFTPD, THBS1, HTR1E, MAP10, RWDD2B, FBXL19, SERPINE1, C3orf14, SLC25A29, RGS17.
- Seed 94: RWDD2B, SFTPD, PDK4, IFT46, IBSP, TPT1, AFF1, XRN2,

ADRA2B, G2E3, KDM2B, FBXL19, MAPK15.

- Seed 118: AGAP2, FKBP1B, SCGB3A1, SLC25A29, TNIP3.

In Covid-19:

- Center-based split:*original_shape_SurfaceArea_disease_left*, *original_gldm_DependenceNonUniformity_disease_right*.
- Seed 10: *D_dimers*, *BMI*, *original_shape_Elongation_disease_right*, *CRP*, *original_shape_Maximum2DDiameterSlice_disease_right*, *original_gldm_GrayLevelNonUniformity_disease_right*, *original_firstorder_Range_disease_right*, *original_shape_Elongation_lung_left*, *Lymphocytes*, *original_ngtdm_Busyness_lung_left*, *original_shape_Maximum2DDiameterSlice_disease_left*, *original_glrlm_RunLengthNonUniformity_disease_right*, *original_gldm_DependenceVariance_disease_left*, *original_firstorder_InterquartileRange_disease_left*, *original_glrlm_RunLengthNonUniformity_lung_right*, *original_glcm_MaximumProbability_lung_right*, *original_shape_LeastAxisLength_heart*, *original_shape_MajorAxisLength_disease_left*, *original_glcm_ClusterProminence_lung_right*, *original_firstorder_Variance_disease_left*, *original_gldm_DependenceVariance_disease_right*, *original_firstorder_Maximum_disease_right*.
- Seed 82: *original_glrlm_GrayLevelNonUniformity_disease_right*, *D_dimers*, *original_firstorder_RobustMeanAbsoluteDeviation_lung_right*,

original_ngtdm_Busyness_lung_left, original_firstorder_Variance_disease_left, CRP, BMI.

- Seed 94: original_gldm_DependenceNonUniformity_disease_right, original_firstorder_Kurtosis_lung_right, D_dimers.
- Seed 118: original_gldm_DependenceNonUniformity_disease_right, D_dimers, BMI, original_shape_Maximum2DDiameterSlice_lung_right, CRP, original_firstorder_Minimum_lung_left, original_firstorder_Kurtosis_disease_right, original_firstorder_Variance_disease_left, original_glrlm_RunLengthNonUniformity_disease_left, original_firstorder_Kurtosis_disea original_glcm_MaximumProbability_lung_right, original_shape_Maximum3DDiameter_heart, original_glrlm_RunLengthNonUniformity_lung_right.

In MI:

- Seed 10: AGE, NITR_S, ZSN_A, S_AD_ORIT, zab_leg_02, NA_R_1_n, ant_im, inf_im, TIME_B_S, L_BLOOD, IM_PG_P, FK_STENOK, K_BLOOD, endocr_01, ROE, ritm_ecg_p_01.
- Seed 82: AGE, ANT_CA_S_n, ZSN_A, GEPAR_S_n, O_L_POST, zab_leg_04, R_AB_1_n, NITR_S, inf_im, TIME_B_S, post_im, L_BLOOD.
- Seed 94: AGE, O_L_POST, ZSN_A, B_BLOK_S_n, TIME_B_S, AST_BLOOD, R_AB_1_n, zab_leg_04, post_im, ANT_CA_S_n, L_BLOOD, STENOK_AN, NITR_S, IM_PG_P, INF_ANAM, fibr_ter_05, n_p_ecg_p_08, ritm_ecg_p_02,

$n\_r\_ecg\_p\_08$, ROE, $n\_p\_ecg\_p\_06$, Na_BLOOD, $n\_p\_ecg\_p\_05$, $NA\_R\_1\_n$, inf_im, $nr07$, ASP_S_n, $n\_r\_ecg\_p\_09$, $nr01$, $DLIT\_AG$, $TRENT\_S\_n$, $ritm\_ecg\_p\_04$, $ritm\_ecg\_p\_06$, GB, $n\_p\_ecg\_p\_03$, $nr03$, $n\_r\_ecg\_p\_10$, $endocr\_03$, SVT_POST, zab_leg_01, FK_STENOK, $endocr\_01$, IBS_POST, S_AD_ORIT, $fibr\_ter\_08$, $n\_r\_ecg\_p\_03$, $fibr\_ter\_06$, lat_im, $n\_r\_ecg\_p\_01$.

- Seed 118: AGE, $GEPAR\_S\_n$, $ANT\_CA\_S\_n$, ZSN_A, zab_leg_06, K_SH_POST, $R\_AB\_1\_n$, $post\_im$, $STENOK\_AN$, $ritm\_ecg\_p\_01$, ALT_BLOOD, FIB_G_POST, L_BLOOD, $fibr\_ter\_06$, zab_leg_01, $NITR\_S$, IM_PG_P, $TIME\_B\_S$, ROE, $ritm\_ecg\_p\_04$, GT_POST, FK_STENOK, $n\_p\_ecg\_p\_09$, $NA\_R\_1\_n$, INF_ANAM, $n\_p\_ecg\_p\_06$, MP_TP_POST, $DLIT\_AG$, inf_im, zab_leg_02, $np07$, $n\_p\_ecg\_p\_07$, Na_BLOOD, $n\_r\_ecg\_p\_09$, $n\_p\_ecg\_p\_03$, D_AD_ORIT, $n\_p\_ecg\_p\_01$, $nr04$, $n\_r\_ecg\_p\_05$, $B\_BLOK\_S\_n$, $nr02$, S_AD_ORIT, $fibr\_ter\_02$.

In TCGA:

- Seed 10: CX3CL1, KLK2, PA2G4P4, CDX1, CHGA, FTHL3, CYP11B1, TSHR, OR6C65, SFTPA1, TYR, ACSM2A, C1orf61, SOX17, AGT.
- Seed 82: GFAP, KLK3, BSND, PA2G4P4, TG, CDX1, NR5A1, CHRNA3, AGT, NACAP1, SFTPA1, OR6C65, SOX17, DSC3, AZGP1.
- Seed 94: KLK3, PA2G4P4, FTHL3, UTF1, TSHR, CHGA, BSND, CDX1, SPN, SFTPA1, CYP11B1, AZGP1, PLP1, GPR87, ACSM2A, KRT74, DCLK2, SOX17, AGT, SLC17A3, NDUFA4L2.
- Seed 118: GFAP, PA2G4P4, TSHR, CHGA, CX3CL1, APLN, UTF1, SLC17A3, SFTPA1, AZGP1, CLCNKA, DSC3, AGT, SOX17, CDX1.

S1 Results Tables

RA-MAP		#Feat.	**BA Tr**	**BA Te**	**WP Tr**	**WP Te**	**WR Tr**	**WR Te**	**WF Tr**	**WF Te**
Baselines	No Selection	10107	1.00 CI: [1.00,1.00]	0.57 CI: [0.57,0.57]	1.00 CI: [1.00,1.00]	0.58 CI: [0.58,0.59]	1.00 CI: [1.00,1.00]	0.58 CI: [0.56,0.60]	1.00 CI: [1.00,1.00]	0.57 CI: [0.56,0.58]
Baselines	MV	21	0.85 CI: [0.81,0.87]	0.55 CI: [0.53,0.59]	0.85 CI: [0.82,0.88]	0.56 CI: [0.54,0.6]	0.85 CI: [0.82,0.88]	0.56 CI: [0.54,0.6]	0.85 CI: [0.82,0.88]	0.56 CI: [0.54,0.6]
Baselines	WMV	16	0.87 CI: [0.84,0.89]	0.51 CI: [0.47,0.59]	0.87 CI: [0.85,0.9]	0.52 CI: [0.47,0.6]	0.87 CI: [0.85,0.89]	0.52 CI: [0.47,0.6]	0.87 CI: [0.85,0.89]	0.52 CI: [0.47,0.6]
Baselines	TPOT	NA	0.91 CI: [0.64,1.0]	0.51 CI: [0.4,0.57]	0.94 CI: [0.75,1.0]	0.53 CI: [0.4,0.58]	0.92 CI: [0.67,1.0]	0.53 CI: [0.4,0.58]	0.91 CI: [0.63,1.0]	0.52 CI: [0.4,0.58]
Baselines	Eular Score	NA	0.53 CI: [0.5,0.56]	0.59 CI: [0.5,0.66]	0.54 CI: [0.51,0.57]	0.61 CI: [0.51,0.7]	0.55 CI: [0.52,0.58]	0.61 CI: [0.53,0.58]	0.53 CI: [0.51,0.56]	0.6 CI: [0.5,0.67]
k-Heavy		20.75	0.83 CI: [0.81,0.84]	0.52 CI: [0.48,0.6]	0.84 CI: [0.81,0.85]	0.53 CI: [0.48,0.6]	0.83 CI: [0.81,0.85]	0.53 CI: [0.49,0.6]	0.83 CI: [0.81,0.85]	0.53 CI: [0.49,0.6]
**k-W Heavy**		**9.5**	**0.75 CI: [0.68,0.8]**	**0.64 CI: [0.59,0.66]**	**0.76 CI: [0.68,0.8]**	**0.64 CI: [0.59,0.67]**	**0.75 CI: [0.67,0.8]**	**0.64 CI: [0.58,0.67]**	**0.75 CI: [0.67,0.8]**	**0.64 CI: [0.58,0.67]**

Table S3: Classification results with confidence intervals on the RA-MAP dataset for the baselines and the proposed approaches.

Covid-19		#Feat.	**BA Tr**	**BA Te**	**WP Tr**	**WP Te**	**WR Tr**	**WR Te**	**WF Tr**	**WF Te**
Baselines	No Selection	547	0.70 CI: [0.70,0.70]	0.62 CI: [0.62,0.62]	0.80 CI: [0.79,0.80]	0.75 CI: [0.75,0.76]	0.69 CI: [0.71,0.71]	0.64 CI: [0.63,0.64]	0.72 CI: [0.73,0.73]	0.67 CI: [0.66,0.67]
Baselines	No Ensemble	15	0.74 CI: [0.74,0.75]	0.63 CI: [0.61,0.64]	0.82 CI: [0.82,0.82]	0.76 CI: [0.74,0.76]	0.75 CI: [0.74,0.75]	0.68 CI: [0.67,0.69]	0.77 CI: [0.76,0.77]	0.70 CI: [0.70,0.71]
Baselines	MV	21	0.76 CI: [0.75,0.77]	0.63 CI: [0.61,0.64]	0.83 CI: [0.82,0.84]	0.75 CI: [0.74,0.76]	0.75 CI: [0.73,0.76]	0.67 CI: [0.65,0.69]	0.77 CI: [0.75,0.78]	0.7 CI: [0.68,0.71]
Baselines	WMV	16	0.75 CI: [0.73,0.79]	0.64 CI: [0.56,0.67]	0.83 CI: [0.81,0.85]	0.76 CI: [0.72,0.78]	0.74 CI: [0.73,0.77]	0.67 CI: [0.62,0.74]	0.76 CI: [0.75,0.79]	0.7 CI: [0.65,0.75]
Baselines	TPOT	NA	0.72 CI: [0.66,0.74]	0.66 CI: [0.64,0.67]	0.8 CI: [0.77,0.82]	0.77 CI: [0.76,0.78]	0.72 CI: [0.68,0.74]	0.68 CI: [0.63,0.73]	0.75 CI: [0.71,0.77]	0.71 CI: [0.67,0.75]
Baselines	KEA	29	0.72 CI: [0.69,0.74]	0.65 CI: [0.6,0.71]	0.81 CI: [0.79,0.82]	0.77 CI: [0.74,0.8]	0.73 CI: [0.72,0.74]	0.68 CI: [0.63,0.75]	0.75 CI: [0.74,0.76]	0.71 CI: [0.66,0.77]
k-Heavy		18.4	0.74 CI: [0.72,0.75]	0.62 CI: [0.57,0.65]	0.82 CI: [0.81,0.83]	0.75 CI: [0.72,0.77]	0.74 CI: [0.68,0.78]	0.67 CI: [0.6,0.74]	0.76 CI: [0.71,0.79]	0.7 CI: [0.64,0.75]
**k-W Heavy**		**9.4**	**0.75 CI: [0.71,0.82]**	**0.68 CI: [0.66,0.72]**	**0.82 CI: [0.8,0.87]**	**0.78 CI: [0.75,0.81]**	**0.73 CI: [0.69,0.78]**	**0.69 CI: [0.67,0.73]**	**0.75CI: [0.72,0.8]**	**0.71 CI: [0.7,0.75]**

Table S4: Classification results with confidence intervals on the Covid-19 dataset for the baselines and the proposed approaches.

Covid-19 (Center-wise split)		#Feat.	BA Tr	BA Te	WP Tr	WP Te	WR Tr	WR Te	WF Tr	WF Te
Baselines	No Selection	547	0.66	0.69	0.77	0.79	0.71	0.70	0.73	0.73
	MV	23	0.77	0.63	0.84	0.76	0.76	0.68	0.78	0.7
	WMV	12	0.73	0.67	0.81	0.78	0.73	0.71	0.75	0.73
	TPOT	NA	0.72	0.64	0.8	0.76	0.74	0.68	0.76	0.71
	KEA	29	0.73	0.7	0.82	0.81	0.67	0.64	0.8	0.77
	Physicians	NA	NA	0.67	NA	0.78	NA	0.7	NA	0.64
	GHOST	NA	0.67	0.71	0.78	0.8	0.69	0.73	0.65	0.69
k-Heavy		20	0.72	0.64	0.81	0.78	0.73	0.71	0.75	0.73
**k-W Heavy**		**2**	**0.71**	**0.72**	**0.8**	**0.81**	**0.7**	**0.73**	**0.73**	**0.75**

Table S5: Classification results on the Covid-19 dataset with the center-wise split KEA was designed on, for the baselines and the proposed approaches.

MI		#Feat.	BA Tr	BA Te	WP Tr	WP Te	WR Tr	WR Te	WF Tr	WF Te
Baselines	No Selection	663	0.71 CI: [0.70,0.71]	0.66 CI: [0.66,0.67]	0.72 CI: [0.7,0.72]	0.68 CI: [0.78,0.68]	0.71 CI: [0.70,0.71]	0.67 CI: [0.66,0.67]	0.71 CI: [0.71,0.71]	0.66 CI: [0.67,0.67]
	No Ensemble	20	0.68 CI: [0.68,0.68]	0.66 CI: [0.66,0.66]	0.70 CI: [0.69,0.70]	0.68 CI: [0.67,0.68]	0.67 CI: [0.67,0.68]	0.65 CI: [0.66,0.64]	0.68 CI: [0.68,0.68]	0.65 CI: [0.64,0.66]
	MV	13.5	0.73 CI: [0.72,0.73]	0.68 CI: [0.67,0.69]	0.75 CI: [0.74,0.76]	0.7 CI: [0.69,0.72]	0.72 CI: [0.7,0.74]	0.66 CI: [0.64,0.7]	0.72 CI: [0.71,0.74]	0.66 CI: [0.64,0.69]
	WMV	25.25	0.74 CI: [0.71,0.77]	0.66 CI: [0.64,0.67]	0.76 CI: [0.74,0.78]	0.68 CI: [0.67,0.7]	0.73 CI: [0.71,0.74]	0.65 CI: [0.63,0.68]	0.73 CI: [0.71,0.75]	0.65 CI: [0.63,0.67]
	TPOT	NA	0.84 CI: [0.7,1.0]	0.68 CI: [0.66,0.7]	0.85 CI: [0.73,1.0]	0.7 CI: [0.68,0.72]	0.83 CI: [0.68,1.0]	0.68 CI: [0.67,0.7]	0.83 CI: [0.68,1.0]	0.68 CI: [0.67,0.7]
k-Heavy		19.5	0.73 CI: [0.72,0.73]	0.68 CI: [0.67,0.69]	0.75 CI: [0.74,0.76]	0.71 CI: [0.69,0.72]	0.71 CI: [0.7,0.74]	0.67 CI: [0.64,0.7]	0.71 CI: [0.7,0.74]	0.67 CI: [0.64,0.69]
**k-W Heavy**		**29**	**0.71 CI: [0.69,0.72]**	**0.69 CI: [0.68,0.7]**	**0.73 CI: [0.7,0.75]**	**0.71 CI: [0.7,0.71]**	**0.69 CI: [0.68,0.7]**	**0.67 CI: [0.65,0.68]**	**0.7 CI: [0.69,0.7]**	**0.67 CI: [0.65,0.68]**

Table S6: Classification results with confidence intervals on the MI dataset for the baselines and the proposed approaches.

TCGA		#Feat.	BA Tr	BA Te	WP Tr	WP Te	WR Tr	WR Te	WF Tr	WF Te
Baselines	MV	17	0.82 +/- [0.77,0.88]	0.77 +/- [0.73,0.81]	0.86 +/- [0.83,0.9]	0.83 +/- [0.8,0.85]	0.83 +/- [0.79,0.88]	0.8 +/- [0.76,0.83]	0.84 +/- [0.8,0.89]	0.81 +/- [0.78,0.84]
	WMV	16.5	0.85 +/- [0.76,0.93]	0.79 +/- [0.73,0.85]	0.9 +/- [0.84,0.95]	0.86 +/- [0.82,0.9]	0.87 +/- [0.79,0.94]	0.83 +/- [0.77,0.89]	0.88 +/- [0.8,0.94]	0.84 +/- [0.78,0.9]
	**TPOT**	**NA**	**0.98 +/- [0.92,1.0]**	**0.93 +/- [0.89,0.99]**	**0.98 +/- [0.93,1.0]**	**0.96 +/- [0.92,1.0]**	**0.98 +/- [0.92,1.0]**	**0.96 +/- [0.91,1.0]**	**0.98 +/- [0.92,1.0]**	**0.96 +/- [0.91,1.0]**
	COMBING	27	0.90 +/- [0.87,0.97]	0.81 +/- [0.80,0.83]	0.90 +/- [0.87,0.96]	0.85 +/- [0.84,0.85]	0.88 +/- [0.84,0.96]	0.82 +/- [0.81,0.84]	0.88 +/- [0.85,0.96]	0.83 +/- [0.82,0.85]
k-Heavy		17	0.73 +/- [0.68,0.79]	0.68 +/- [0.65,0.74]	0.78 +/- [0.74,0.83]	0.74 +/- [0.71,0.8]	0.72 +/- [0.65,0.79]	0.68 +/- [0.62,0.75]	0.73 +/- [0.66,0.8]	0.7 +/- [0.64,0.77]
**k-W Heavy**		**16.5**	**0.96 +/- [0.91,1.0]**	**0.83 +/- [0.82,0.85]**	**0.97 +/- [0.93,1.0]**	**0.89 +/- [0.88,0.9]**	**0.95 +/- [0.87,1.0]**	**0.87 +/- [0.81,0.89]**	**0.95 +/- [0.88,1.0]**	**0.87 +/- [0.83,0.89]**

Table S7: Classification results with confidence intervals on the TCGA dataset for the baselines and the proposed approaches.

S2 Results Figures

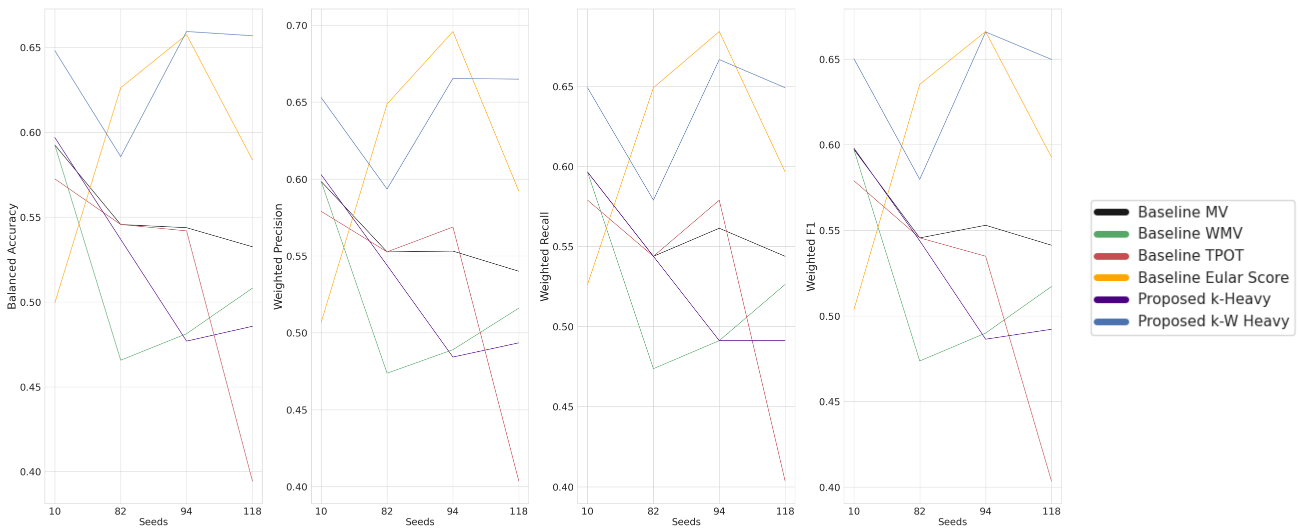

Figure S2: Classification performance on the RA-MAP dataset. We compare the results of the proposed method, k-W Heavy with several state-of-the-art ensembling feature selection and autoML approaches over different seeds.

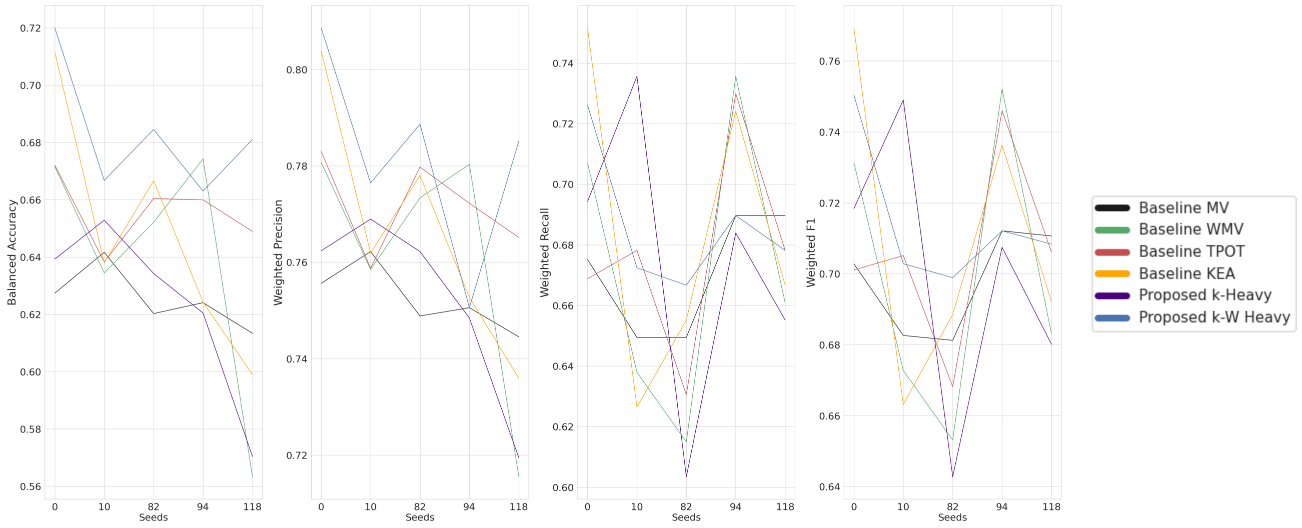

Figure S4: Classification performance on the Covid-19 dataset. We compare the results of the proposed method, k-W Heavy with several state-of-the-art ensembling feature selection, autoML, and task-specific approaches over different seeds.

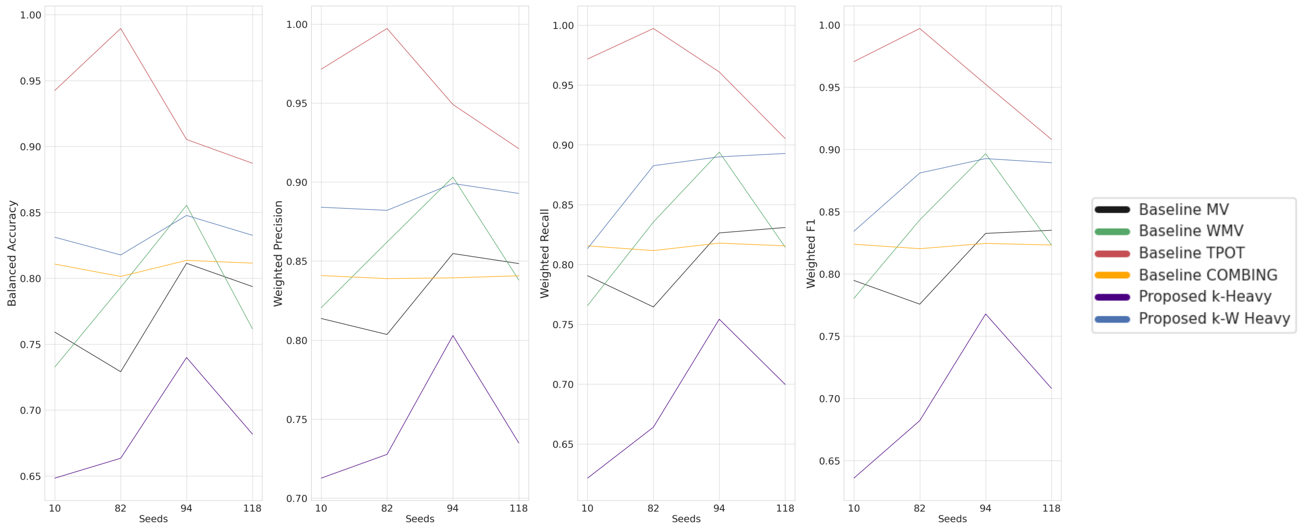

Figure S5: Classification performance on the TCGA dataset. We compare the results of the proposed method, k-W Heavy with several state-of-the-art ensembling feature selection, autoML, and task-specific approaches over different seeds.

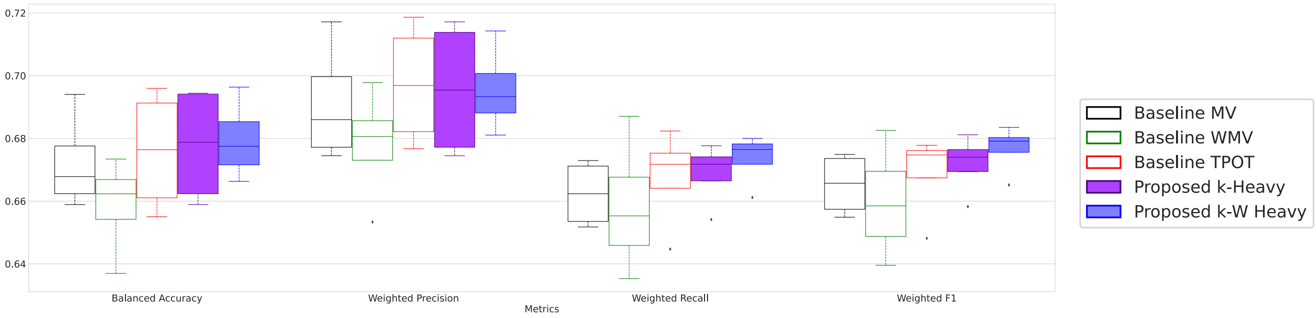

(a) MI

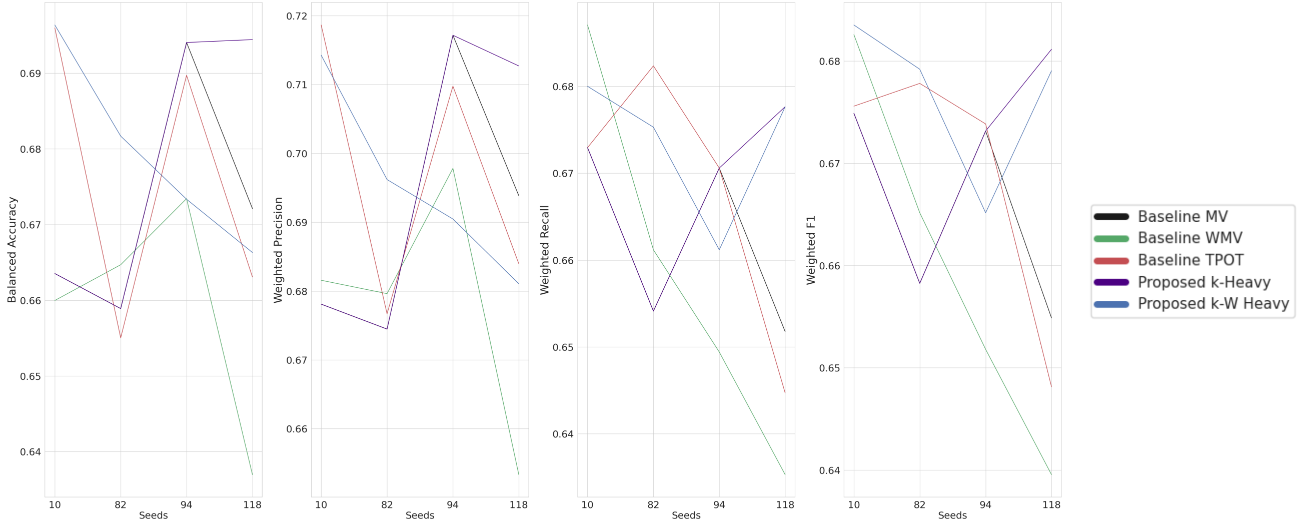

Figure S7: Classification performance on the MI dataset. We compare the results of the proposed method, k-W Heavy with several state-of-the-art ensembling feature selection, and autoML approaches over different seeds.

S3 TCGA: Signature Size Constraint

It is to note that the performances reported for the maximal number of features in Figure S9 are slightly higher than the ones reported with fewer features in Section 3. This is explained by the fact that the results on cross-validation with 21 features were lower or did not respect the constraints imposed by the proposed model selection criteria.

S4 Selection Rule Influence

We present in Figure S10 a plot of the BA performance given different rules that can be defined from the definition described in Methods, Section S0.3. In this regard, we consider the metrics defined in Methods, Section S0.1, the lower threshold 0 the upper thresholds 0.05 and 0.1, and the functions "$-$" and "$|-|$" where $\forall m \in \mathcal{M},\ \forall r \in \mathcal{R},\ |-|(m(r)) = |m^{Tr}(r) - m^{Val}(r)|$. The set of rules we study is given by the set of combinations of the components mentioned above.

In Figure S10, we report the performance on the four datasets for the proposed $k-WHeavy$ and the $TPOT$ autoML baseline to see the influence of the rules on the proposed pipeline compared to another autoML framework. On each subfigure, the rules are ordered according to the function first, then the upper threshold, and finally, the metrics used.

We can first observe that k-W Heavy is more sensitive to the considered rule as this is an intrinsic property of the proposed pipeline. Notwithstanding, even for TPOT, we report a maximal difference on BA of 0.23 for the same seed. Besides, over the different datasets, different rules overperform, with some datasets (Covid-19 and

RA-MAP) presenting a lesser resilience to rule change. However, this trend is likely strongly correlated to overfitting, with some rules being more prone to it. In this case, a rule that usually allows high performance might fail on a dataset such as RA-MAP, high-dimensional and noisy because it will not generalize on the test. In particular, rules relying on one metric only are more likely to overfit.

We observe that there is a symmetry left/right in the subfigures associated to TPOT meaning that the function has very little influence on the performance. However, in the case of the K-W Heavy method, there is a more significant discrepancy. Then, the influence of the threshold can be seen by the presence of small continuous horizontal segments while the importance of the metric is seen by a series of dots of the same performance regularly separated. In this case, according to the seed, dataset and method considered, the combination threshold and metrics have an important impact. On RA-MAP with the k-W Heavy method, some metrics enable to get higher performance whatever the function or threshold while with TPOT very few specific metrics offer better performance with a specific threshold. Some experiments do not seem to present a clear pattern allowing to identify a parameter performing well in any combination, see for instance Figure S10b. Notwithstanding, there is always a combination performing well whatever the seed.

This highlights the importance of the rule considered for model selection and proves the interest of pursuing a broader study of the formalism introduced in Section S0.3.

S5 Sample Size Influence with Varying Test Sets

This section offers additional experiments to the sample size experiments presented in the main document (Section S0.6). Here, we investigate the sample size effect when the size of the test set is varying as the same time as the training set's one. This experiment models the effect of sample size on estimating the generalizability of one's results according to the considered sample size.

First, note that, as expected, the classification performance are less stable when the test sets vary. Also, the dependence on the seed is stronger, denoting a lesser generalizability. Finally, the plateau is harder to determine and to correlate with the one of the density. However, this experiment enables us to show the risk of bias when conducting a machine learning study without enough samples while emphasizing the interest of the study of the density. Indeed, studying the convergence of the density would allow to ensure the number of samples considered is sufficient.

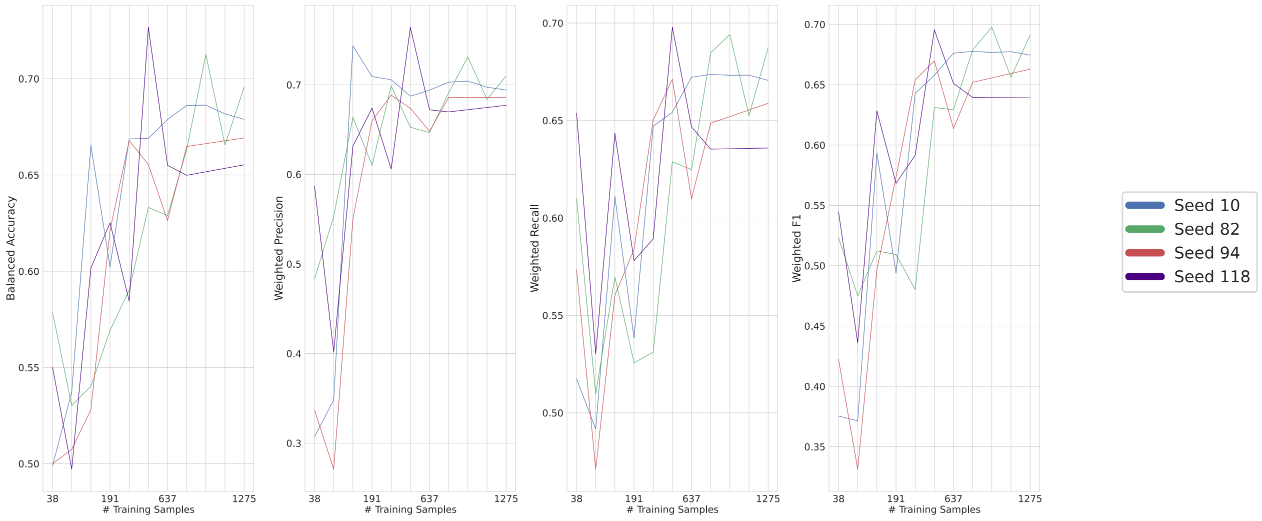

Figure S11: Sample Size Influence on the MI dataset with changing test sets. We study the results of the proposed approach with different sizes of training sets. We measure the classification performance in the co-selection graph of the subgraph selected by k-W Heavy. Here, the test set and its sizes are modified depending on the sample size.

References

[1] Bastien Caba, Dawei Liu, Aurélien Lombard, and Natasha et al. Novikov. Machine learning-based classification of acute versus chronic multiple sclerosis lesions using radiomic features from unenhanced cross-sectional brain mri (4121). *Neurology*, 2021.

[2] Yvan Saeys, Thomas Abeel, and Yves Van de Peer. Robust feature selection using ensemble feature selection techniques. In *Joint European conference on machine learning and knowledge discovery in databases*. Springer, 2008.

[3] Trang T Le, Weixuan Fu, and Jason H Moore. Scaling tree-based automated machine learning to biomedical big data with a feature set selector. *Bioinformatics*, 2020.

[4] Enzo Battistella, Maria Vakalopoulou, Roger Sun, and Theo et al. Estienne. Combing: Clustering in oncology for mathematical and biological identification of novel gene signatures. *IEEE/ACM transactions on computational biology and bioinformatics*, 2021.

[5] Iuliia Biliavska, Tanja A Stamm, Jose Martinez-Avila, and Thomas WJ et al. Huizinga. Application of the 2010 acr/eular classification criteria in patients with very early inflammatory arthritis: analysis of sensitivity, specificity and predictive values in the save study cohort. *Annals of the rheumatic diseases*, 2013.

[6] Guillaume Chassagnon, Maria Vakalopoulou, Enzo Battistella, and Stergios et al. Christodoulidis. Ai-driven quantification, staging and outcome prediction of covid-19 pneumonia. *Medical image analysis*, 2021.

[7] Enzo Battistella, Maria Vakalopoulou, Nikos Paragios, and Eric Deutsch. Ghost: Graph higher-order similarity transformation for classification. 2022.

[8] Shushan Toneyan, Ziqi Tang, and Peter K. Koo. Evaluating deep learning for predicting epigenomic profiles. *Nature Machine Intelligence*, 2022.

[9] Thiloshon Nagarajah and Guhanathan Poravi. A review on automated machine learning (automl) systems. In *2019 IEEE 5th International Conference for Convergence in Technology (I2CT)*, 2019.

[10] Mickael Leclercq, Benjamin Vittrant, Marie Laure Martin-Magniette, and Marie Pier Scott Boyer et al. Large-scale automatic feature selection for biomarker discovery in high-dimensional OMICs data. *Frontiers in Genetics*, 2019.

[11] Maren Mahsereci, Lukas Balles, Christoph Lassner, and Philipp Hennig. Early stopping without a validation set. *arXiv preprint arXiv:1703.09580*, 2017.

[12] Lefteris Koumakis. Deep learning models in genomics; are we there yet? *Computational and Structural Biotechnology Journal*, 2020.

[13] Charalampos Tsourakakis, Francesco Bonchi, Aristides Gionis, Francesco Gullo, and Maria Tsiarli. Denser than the densest subgraph: extracting optimal quasi-cliques with quality guarantees. In *Proceedings of the 19th ACM SIGKDD international conference on Knowledge discovery and data mining*, 2013.

[14] Maximilien Danisch, T-H Hubert Chan, and Mauro Sozio. Large scale density-friendly graph decomposition via convex programming. In *Proceedings of the 26th International Conference on World Wide Web*, 2017.

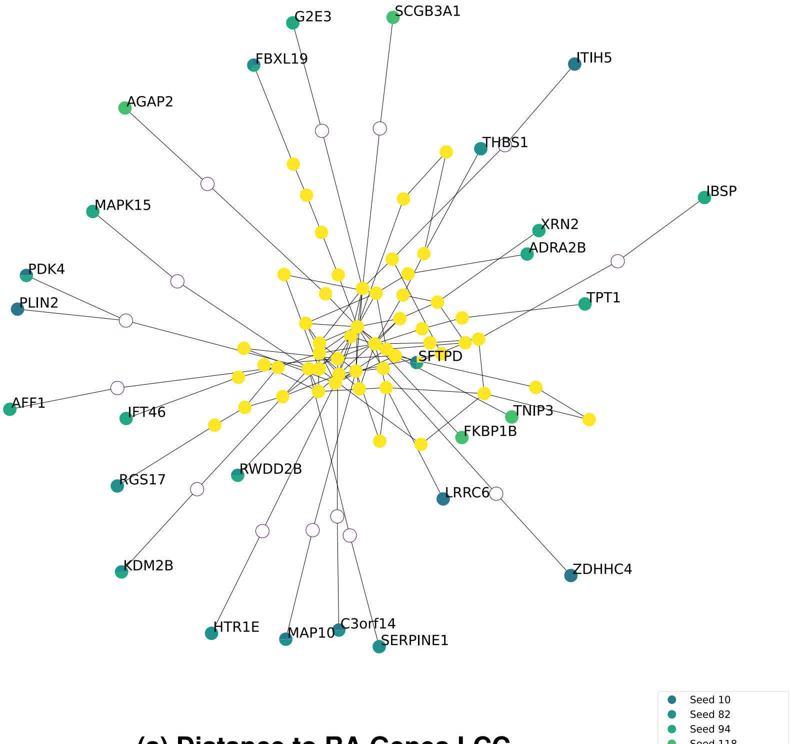

(a) Distance to RA Genes LCC.

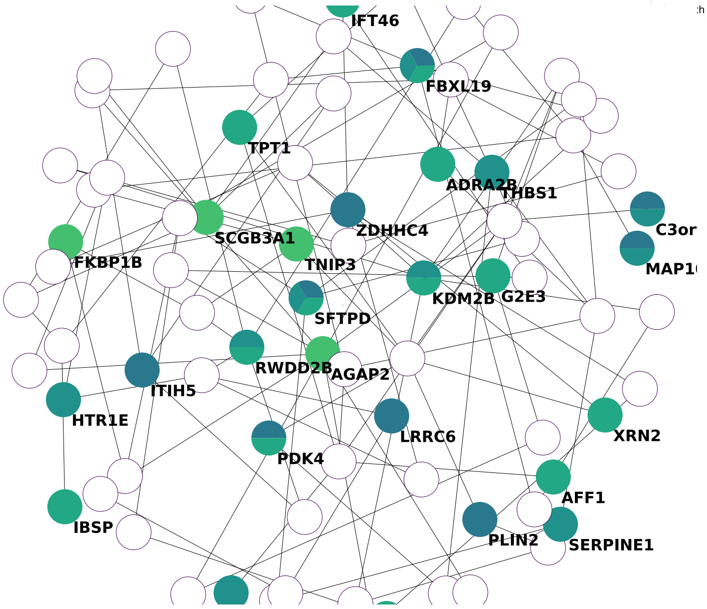

(b) Distance between the signatures of different seeds.

Figure S3: Network representation of the biomarkers selected by the proposed k-W Heavy approach for RA-MAP. We position the 27 genes selected by the k-W Heavy method in the PPI and compute the shortest distances between: **(a)** the genes selected by the proposed k-W heavy method and the genes of the largest connected component (LCC) for RA and **(b)** between the genes selected with different seeds. In **(a)**, the average distance is low, attesting to the biological relevance of the selected genes for RA. In **(b)**, the selected genes are distant from one another, ensuring the diversity of the biological processes they cover.

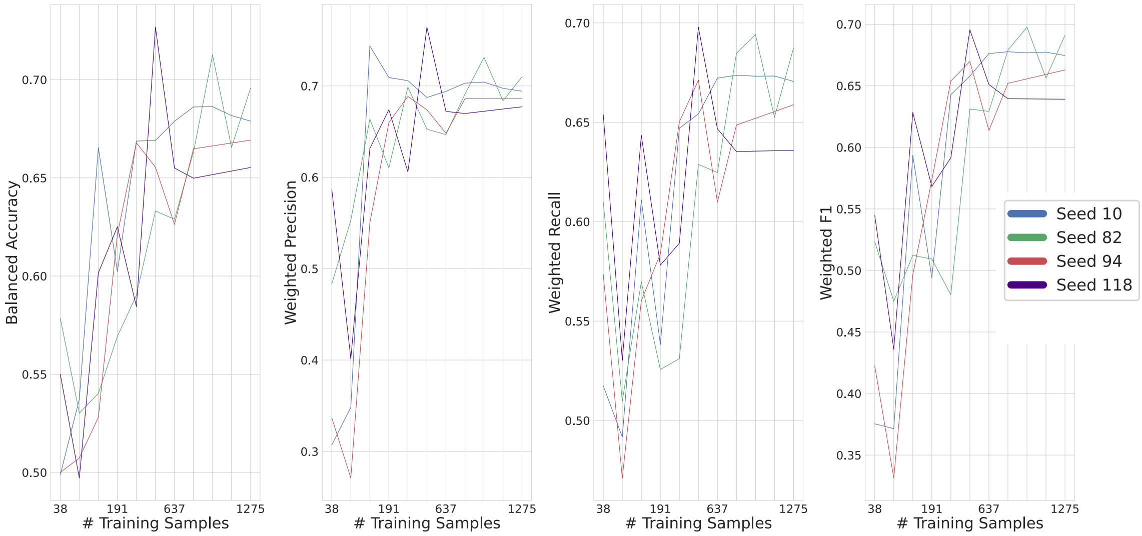

(a) Classification performance of the k-W Heavy algorithm for different training sample sizes.

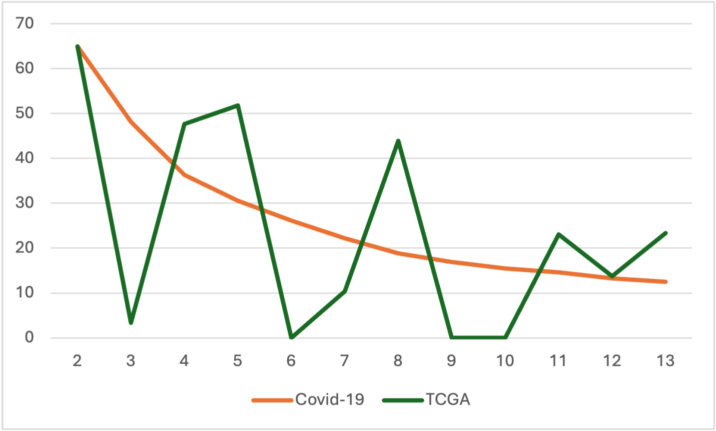

(b) Density of the selected features subgraph for different training sample sizes.

Figure S8: Sample Size Influence on the MI dataset. We study the results of the proposed k-W Heavy with different sizes of training sets, including from 38 to 1275 training samples. We measure the classification performance and the density in the co-selection graph of the subgraph selected by k-W Heavy. The test set remains the same for all sample sizes. The results are averaged over the different seeds. We observe that both the classification performance and the density reach a plateau at 637 samples, proving the correlation between density and classification performance.

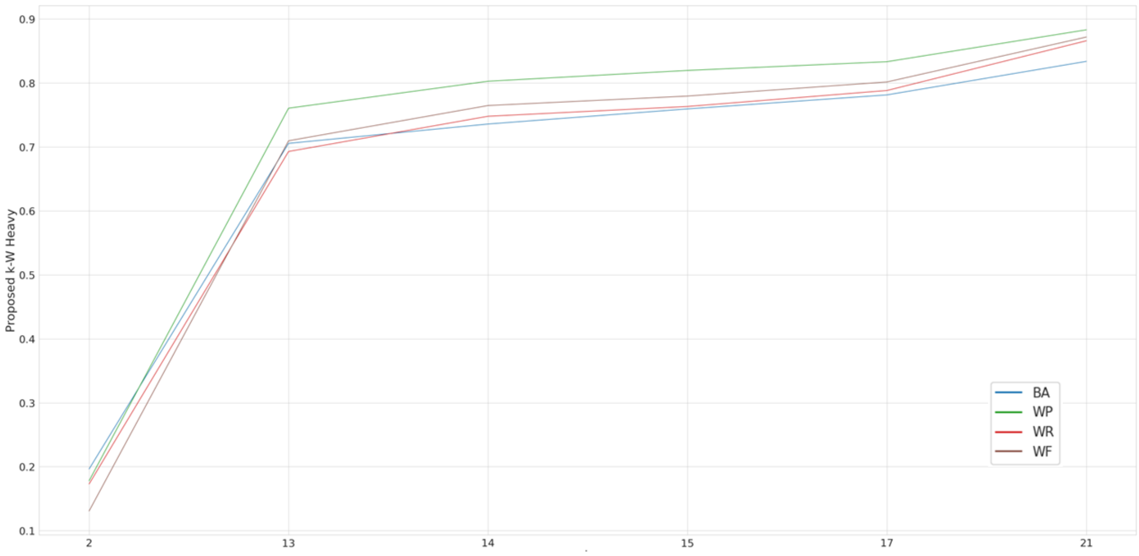

Figure S9: Influence of the number of features on the proposed k-W Heaviest approach for the TCGA dataset.

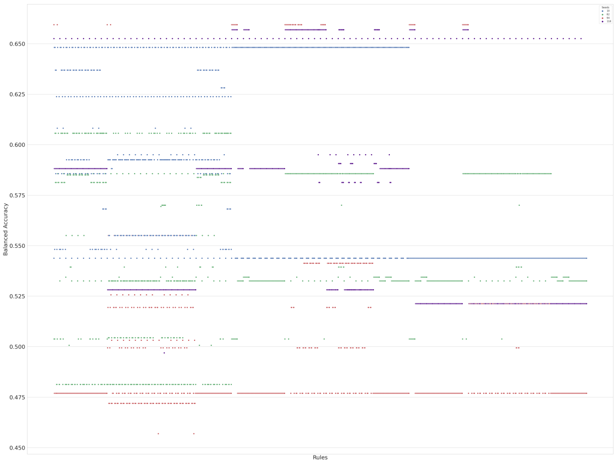

(a) RA-MAP: Proposed k-W Heavy

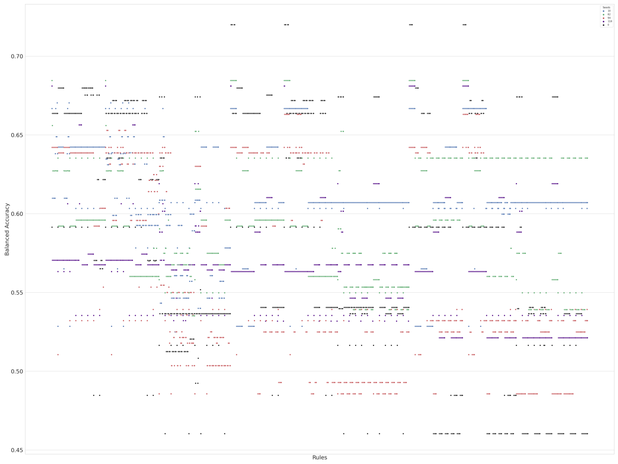

(b) Covid-19: Proposed k-W Heavy

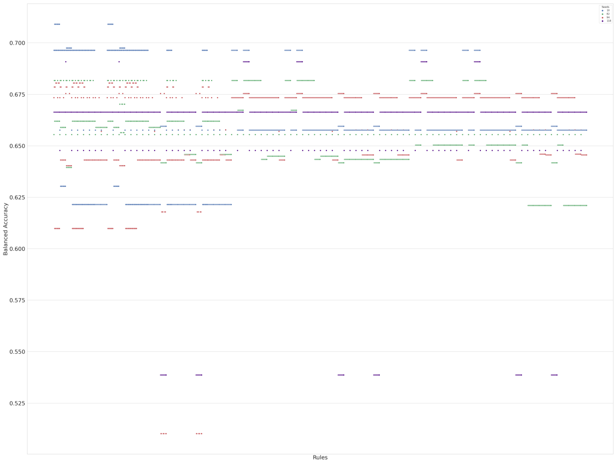

(c) MI: Proposed k-W Heavy

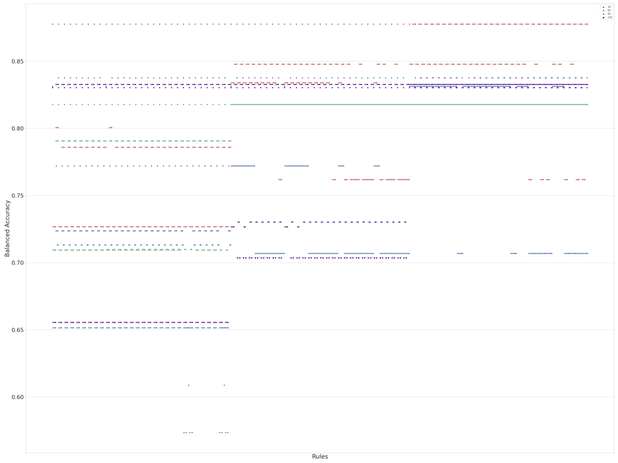

(d) TCGA: Proposed k-W Heavy

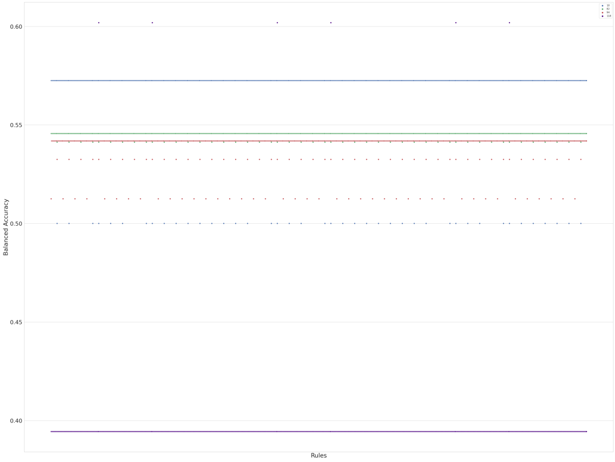

(e) RA-MAP: TPOT Baseline

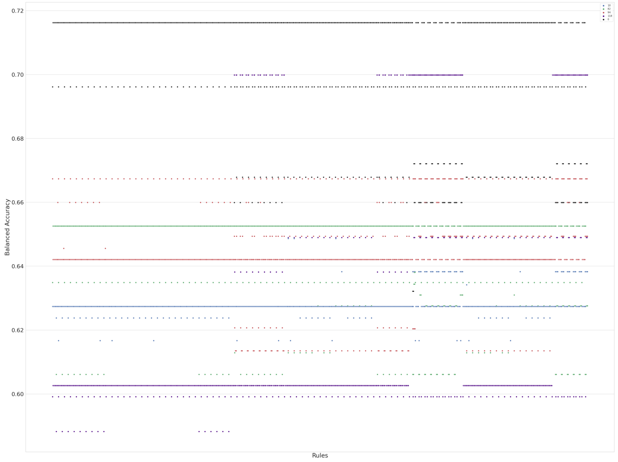

(f) Covid-19: TPOT Baseline

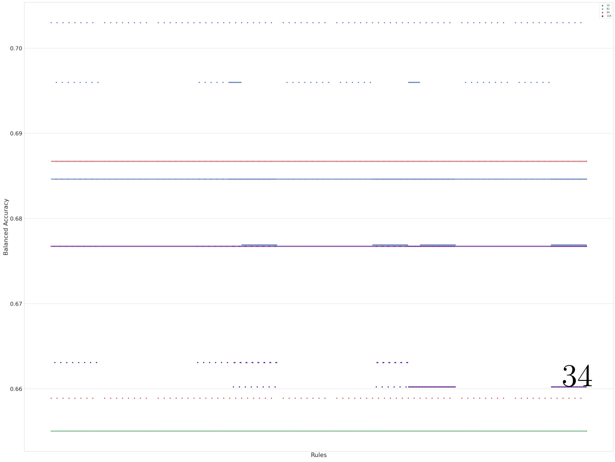

(g) MI: TPOT Baseline

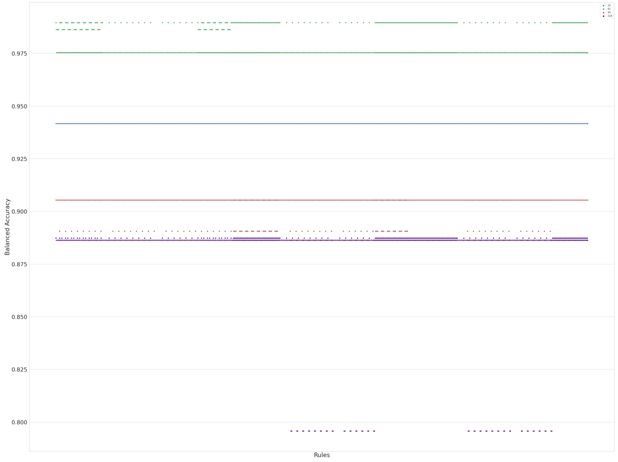

(h) TCGA: TPOT Baseline

Figure S10: Classification performance on the RA-MAP dataset. We compare the results of the proposed method k-W Heavy with the state-of-the-art autoML approach TPOT. The different colors represent the different seeds.
